# Supplementary material for: A comprehensive evaluation of module detection methods for gene expression data
Source: Nat Commun. 2018 Mar 15;9:1090. doi: 10.1038/s41467-018-03424-4 (PMC5854612; doi:10.1038/s41467-018-03424-4)
Supplement: Supplementary file 1 — Supplementary Information(PDF 23262 kb) [file 41467_2018_3424_MOESM1_ESM.pdf]

Supplementary material for

A comprehensive evaluation of module detection methods for gene expression data

Saelens et al.

Contents

Supplementary Tables 2

Supplementary Figures 6

Supplementary Note 1: Measures for comparing overlapping modules 30

Supplementary Note 2: Module detection methods 36

1 Clustering 36
2 Decomposition 42
3 Biclustering 43
4 Direct network inference 46
5 Iterative network inference 48

Supplementary Note 3: Alternative similarity measures 48

Supplementary Note 4: Previous evaluation studies 50

Supplementary References 51

Supplementary Tables

Supplementary Table 1: Overview of freely available tools for module detection. The marker of a method is filled if this tool was used for the evaluation of that particular method. Tools with a graphical user interface (GUI) can be both local, usually using the local computing resources, or web-based, using the computing resources of the server.

Name	Methods	Availability	Website/References
**Clustering**			
flame	A	Command-line	[1]
wgcna	L M	R	[2, 3]
mcl	G	Command-line	[4]
scikit-learn	I H M K F T	Python	[8]
apcluster	H	R	[5, 6]
ELKI	H B M M F T V	Java, **GUI (local)**	*elki.dbs.ifi.lmu.de* [7]
cluster	B M P	R	[9]
nbclust	D N	R	[22]
cluto	M F	Command-line	
matlab	M E F	Matlab	
GenePattern	M E	**GUI (web)**	*genepattern.org* [13]
BicAT	M F	Command-line, **GUI (local)**	*tik.ee.ethz.ch/sop/bicat* [11]
gitools	M	**GUI (local)**	*gitools.org* [15]
Sleipnir	M	Command-line	[10]
weka	M F T	Java	[12]
babelomics	M F Q	**GUI (web)**	*babelomics.org* [14]
transitivity clustering	M J	Command-line, **GUI (local,web)**	*transclust.compbio.sdu.dk* [16]
Expander	M S	Command-line, **GUI (local)**	*acgt.cs.tau.ac.il/expander* [17–19]
orange	M	**GUI (local), Python (visual programming)**	*orange.biolab.si/*
scipy	M	Python	[20]
fastcluster	M	Python, R	[21]
GENE-E	M	**GUI (local)**, R, Java	*broadinstitute.org/cancer/software/GENE-E*
fclusttoolbox	C	Matlab	
mfuzz	C	R/Bioconductor	[23]
kohonen	E	R	[24]
hybridHclust	O	R	
clValid	Q	R	[26]
densityClust	R	R	[25]
fpc	T	R	
clues	U	R	[27]
ConsensusClusterPlus		R/Bioconductor	[32]
Lemon-Tree		Java	[29]
mclust		R	[30, 31]
Bayesian Hierarchical Clustering		R	[28]

Name	Methods	Availability	Website/References
**Decomposition**			
**Matrix decomposition**			
fastICA	A B C	R	
PCA and ICA	A B C E	Matlab	
scikit-learn	A B C E	Python	[8]
FastICA for JAVA	A B	Java	
mixOmics	D	R	[33]
stats	E	R	
nimfa		Python	[34]
**Postprocessing**			
fdrtool	A B D E	R	[35, 36]

**Biclustering**			
scikit-learn	A	Python	[8]
biclust	A F H	R	[37]
isa2	B	R	[39]
BicAT	B H	Command-line, **GUI (local)**	*tik.ee.ethz.ch/sop/bicat* [11]
FABIA	E	R/Bioconductor	[38]
QUBIC	C	R, Command-line, **GUI (web)**	*sbl.bmb.uga.edu/ maqin/bicluster/* [40]
BiCluE	D	Command-line	[41]
msbe	G	Command-line	[42]
ELKI	H	Java, **GUI (local)**	*elki.dbs.ifi.lmu.de* [7]
opsm	I	Command-line	[43]
BicMix		Command-line	
cMonkey		R	
BiBench		Python	[44]
blockcluster		R	[45]

**Network inference followed by graph clustering**			
**Direct network inference**			
GENIE3	A	R	[47]
GP-DREAM	A B D	**GUI (web)**, Command-line	*dream.broadinstitute.org* [46]
minet	B	R/Bioconductor	[49]
CLR	B	Matlab	[48]
TIGRESS	D	Matlab	[50]
ARACNE		Command-line, **GUI (local)**	*califano.c2b2.columbia.edu/aracne* [51]
**Graph clustering**			
mcode		**GUI (local)**	*baderlab.org/Software/MCODE* [52]
transitivity clustering		Command-line	[16]
mcl		Command-line	[4]
apcluster		R	[5]
**Module network inference**			
Lemon-Tree		Java	[29]

Name	Methods	Availability	Website/References
**Iterative network inference**			
GP-DREAM	A	**GUI (web)**, Command-line	*dream.broadinstitute.org* [46]
merlin	A	Command-line	[53]
Genomica	B	**GUI (local)**	[54]

Supplementary Table 2: Overview of freely available tools for the visualization of co-expression modules.

**Visualization of modules**		
d3heatmap	R	Interactive heatmaps
plotly	R, Python, **GUI (web)**	
gitools	**GUI (local)**	Interactive heatmaps with additional annotations
pheatmap	R	Heatmaps with additional annotations
ComplexHeatmap	R/Bioconductor	
gplots	R	
GENE-E	**GUI (local)**, R, Java	
matplotlib	Python	
Heatplus	R/Bioconductor	
Furby	**GUI (local)**	Interactive visualization of biclusters and their relationships
BicOverlapper	**GUI (local)**	
ExpressionView	**GUI (local)**	

Supplementary Table 3: Overview of freely available tools for the functional interpretation of co-expression modules, using biological functional terms such as Gene Ontology, pathway analysis or disease associations.

**Functional interpretation of genes within modules**		
gitools	**GUI (local)**	Enrichment for GO terms and KEGG pathways
Enrichr	**GUI (web)**	Enrichment for a vast variety of functional, pathway or disease related gene sets
Enrichment Map	**GUI (local)**	Cytoscape plug-in to visualize results of module enrichment
FGNet	R/Bioconductor	Interpreting and visualizing functional enrichment in gene networks
ReactomePA	R/Bioconductor	Pathway analysis of Reactome pathways
ConsensusPathDB	**GUI (web)**	Pathwaw enrichment
AmiGO	**GUI (web)**	GO enrichment
ReViGO	**GUI (web)**	Dimensionality reduction of enrichment results
gProfiler	**GUI (web)**	Enrichment for GO terms, KEGG pathways and OMIM disease associations
gostats	R	GO enrichment while taking into account the structure of the GO graph
topGO	R/Bioconductor	
clusterProfiler	R/Bioconductor	Assess enrichment of modules for GO terms, pathways, disease associated genes and custom gene sets
DAVID	**GUI (web)**	
MSigDB	**GUI (web)**	Assess enrichment of modules for GO terms, pathways and disease associated genes
GSEA	**GUI (local)**	

Supplementary Table 4: Overview of freely available tools to infer and visualize regulatory module networks.

Inferring and visualizing regulatory module networks		
GP-DREAM	**GUI (web)**	State-of-the-art tools for inferring regulatory networks
Cytoscape	**GUI (local)**	Visualization of (regulatory) networks
Lemon-Tree	Command-line	Infer and visualize the regulation of modules as decision trees

Supplementary Table 5: Overview of freely available tools for parameter estimation of module detection methods.

Parameter estimation using cluster validity indices or external measures		
nbclust	R	**Internal**: Average silhouette width, Calinski-Harabasz index, Davis-Bouldin index and many more
scikit-learn	Python	**Internal**: Average silhouette width
clValid	R	**External**: BHI<br>**Internal**: Dunn index
clusterCrit	R	**Internal**: Average silhouette width, Calinski-Harabasz index, Davis-Bouldin index and many more
clv	R	**Internal**: Davis-Bouldin index and many more
Cluster Validity Analysis Platform	Matlab	**Internal**: Calinski-Harabasz index, Davis-Bouldin index and many more

Supplementary Figures

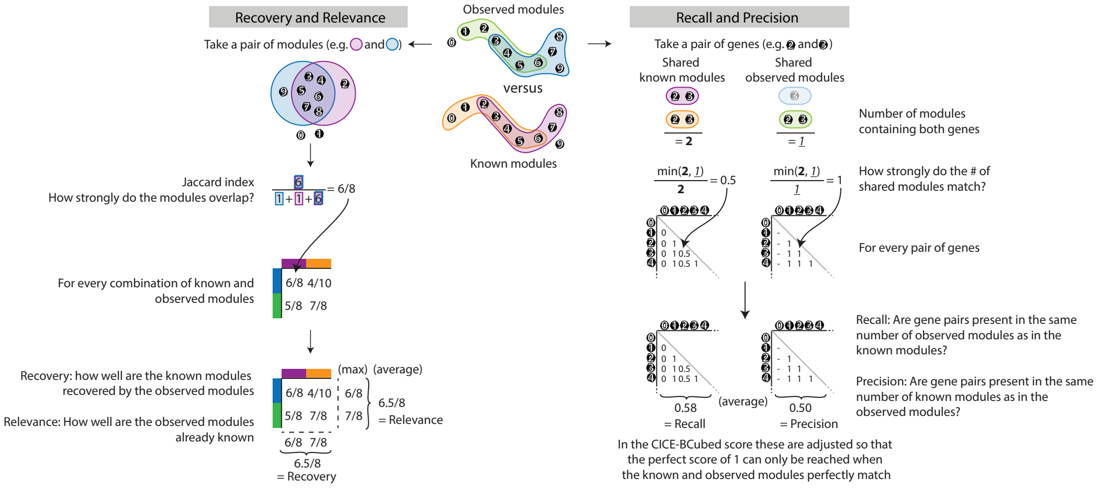

Supplementary Figure 1: Illustration of the four main scores used in this study.
Each score assesses the similarity between a set of known modules and a set of observed modules. The recovery and relevance will try to match individual modules between the two sets using the Jaccard index, a measure of overlap between two mathematical sets. The recovery tries to match known modules with observed modules, while the relevance tries to match observed modules with known modules. The recall and precision scores will compare the number of times a pair of genes is together present in observed modules versus those in known modules. The recall determines whether a pair of genes is present in at least the same number of modules in the known modules as in the observed modules, and vice-versa for the precision.

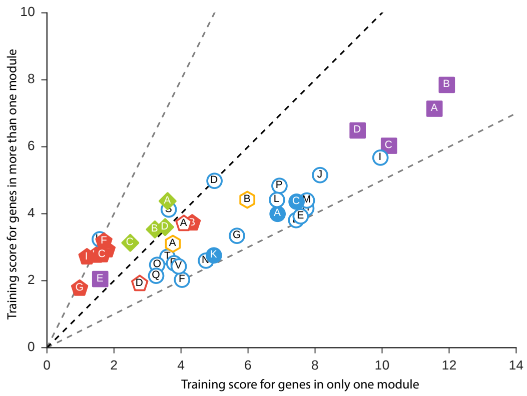

Supplementary Figure 2: Bias of module detection methods towards genes within one module. Shown are training scores (using the harmonic mean of only the Recall and Precision as these are gene-based scores) for genes present in only one module (x-axis) versus genes in more than one module (y-axis) in the minimal co-regulation module definition. Dotted lines represent an equal score (black) or a two-fold higher score (grey). The shape of a method is filled if it can detect overlapping modules (**Supplementary Note 2**). We found that most clustering and decomposition methods are better at correctly grouping genes present in one module, while biclustering methods are slightly biased towards genes in more than one module. Despite this, decomposition methods still outperform every other category both at genes within one module as well as for genes in multiple modules.

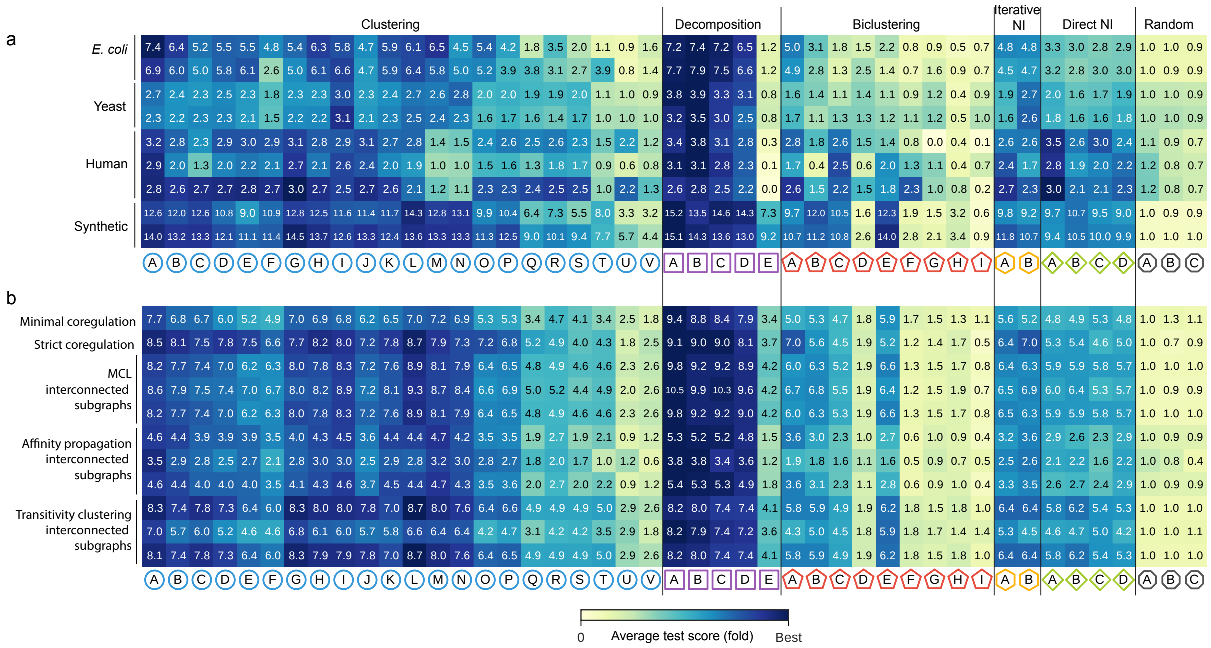

Supplementary Figure 3: Average test scores across different datasets (top) and module definitions (bottom).
(a) While the performance of individual methods is variable across the different datasets (y-axis), the relative performance of the top methods within every category remains relatively stable. For example, in none of the datasets do biclustering methods outperform decomposition methods, although some biclustering methods do perform relatively well on human and synthetic datasets. **(b)** The relative performance of the different methods is very stable between different module definition.

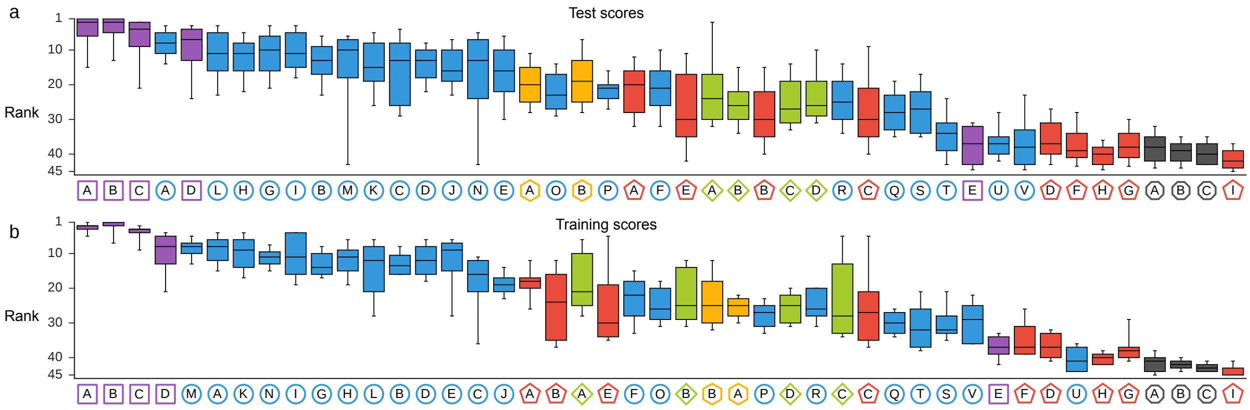

Supplementary Figure 4: Variability of performance for all module detection methods.
We calculated score for every combination of gold standard regulatory network, module definition, training datasets and (in case of test scores) test dataset. Shown are the distributions of the ranks of each method along each of these combinations, where every combination was weighted so that each of the three module definitions (minimal co-regulation, strict co-regulation and interconnected subgraphs) and each of the four organisms (*E. coli*, yeast, human and synthetic) had equal weight. Whiskers denote 10% and 90% weighted percentiles, while the box denotes 25% and 75% percentiles. (**a**) Methods are ordered according to their average test score. (**b**) Methods are ordered according to their average training score.

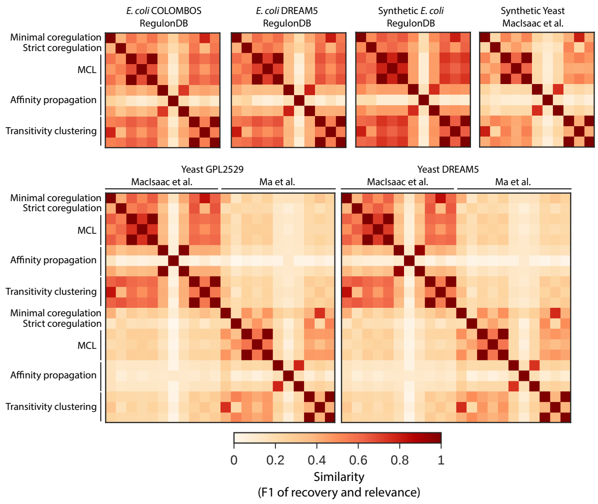

Supplementary Figure 5: Similarity between the known modules when using different module definitions and regulatory networks.
Similarity between modules was calculated using the recovery and relevance scores, which assess how well the modules from one set can be matched to the modules of the other set and vice-versa.

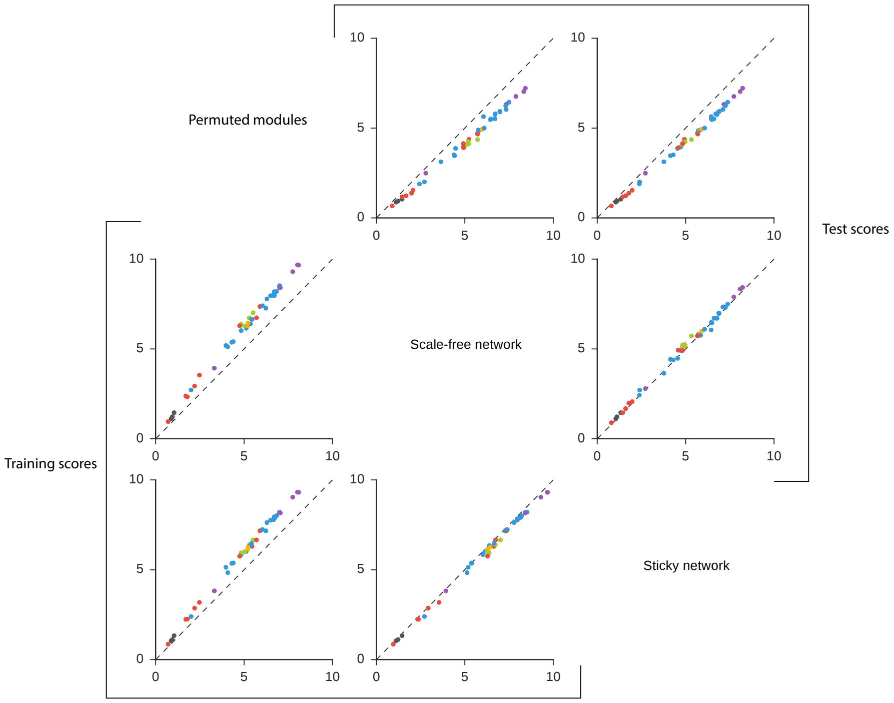

Supplementary Figure 6: Comparison of different module randomization strategies for score normalization. Apart from permuted modules (where starting from a set of known modules, every gene is mapped to a random permutation of all genes) we also looked at two alternative randomization strategies which work on the level of the gold standard regulatory network. We found that using either a random scale-free network or a sticky network (in which each gene and transcription factor keeps the same in- and out-degree) to normalize the score had little effect on the resulting ranking of the different methods.

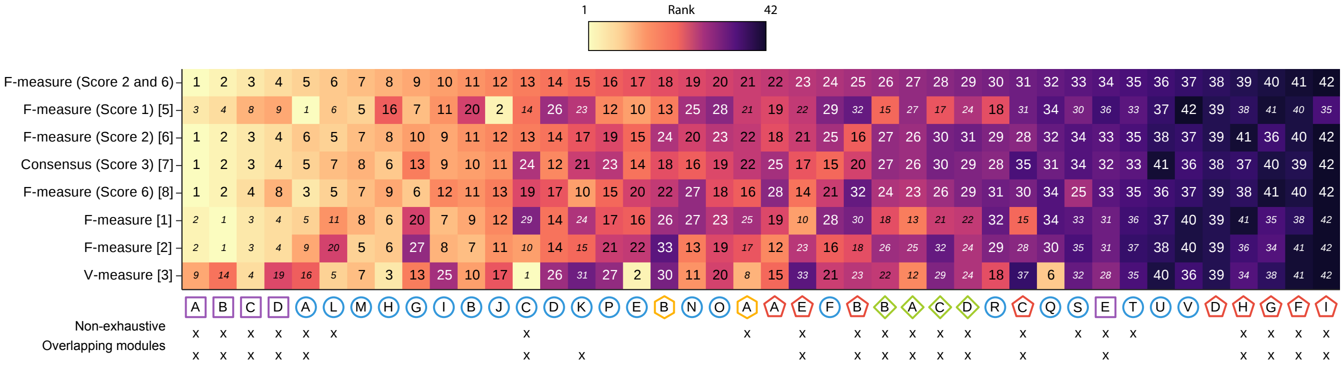

Supplementary Figure 7: Comparison of method ranks across different scoring metrics. Each score (y-axis) assesses the correspondence between a set of observed and known modules. Some scores have some difficulties with handling overlapping and/or non-exhaustive module assignment (**Supplementary Note 1**). Ranks which are potentially unreliable, if also the method detects non-exhaustive and/or overlapping modules (bottom), are therefore shown in a smaller font. Despite this, we found that the overall ranking of most methods was similar between most scores.

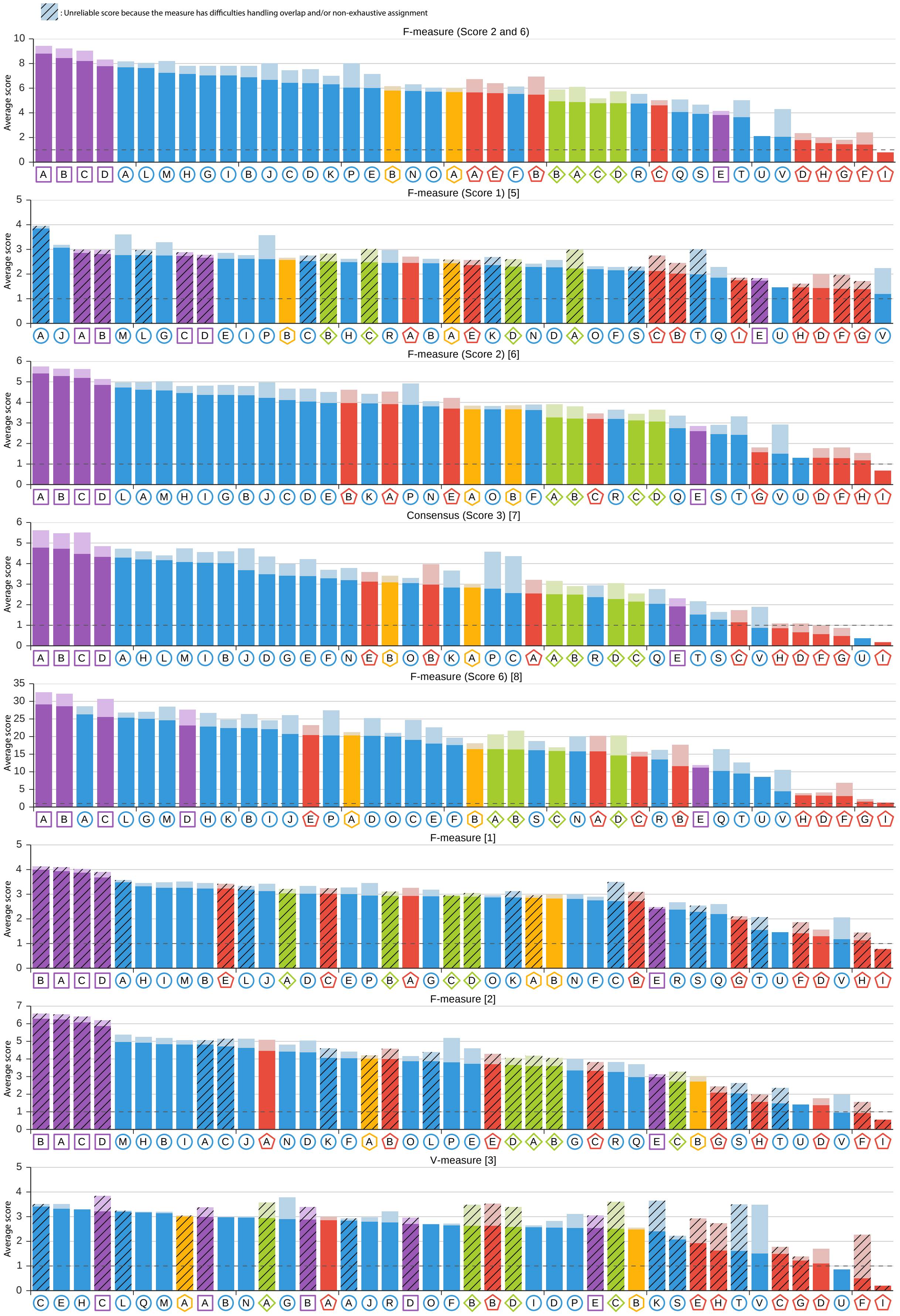

Supplementary Figure 8: Comparison of method test and training scores across different scoring metrics. Each score (y-axis) assesses the correspondence between a set of observed and known modules (**Supplementary Note 1**). Some scores have some difficulties with handling overlapping and/or non-exhaustive module assignment (**Supplementary Note 1**). Scores which are potentially unreliable, if also the method detects non-exhaustive and/or overlapping modules, are therefore shown with a diagonal pattern. Methods are ordered according to their average test score.

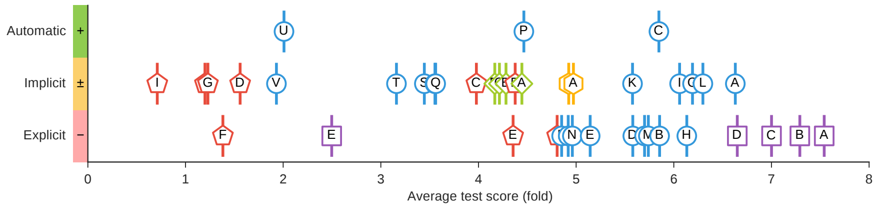

Supplementary Figure 9: Comparison between different ways of estimating the number of modules.
One of the most important parameters for most module detection methods are those influencing the number of modules found in the dataset. There are three ways a method can determine the number of modules: (i) explicitly, by retrieving a fixed number of modules determined by the user, (ii) implicitly, by using other parameters determined by the user to estimate the number of modules and (iii) automatically, by determining the number of modules independent of parameters. Implicitly or automatically determining the number of methods can therefore allow methods to better adapt to individual characteristics of a dataset, although it can also lead to a suboptimal number of modules when compared with a given gold standard. We found that among clustering methods those that implicitly estimated the number of methods performed better than their explicit counterparts. However, implicit or automatic module number estimation is (among current methods) not mandatory for optimal performance, as all decomposition methods have a user parameter for the number of components (and thus the number of modules) within the data.

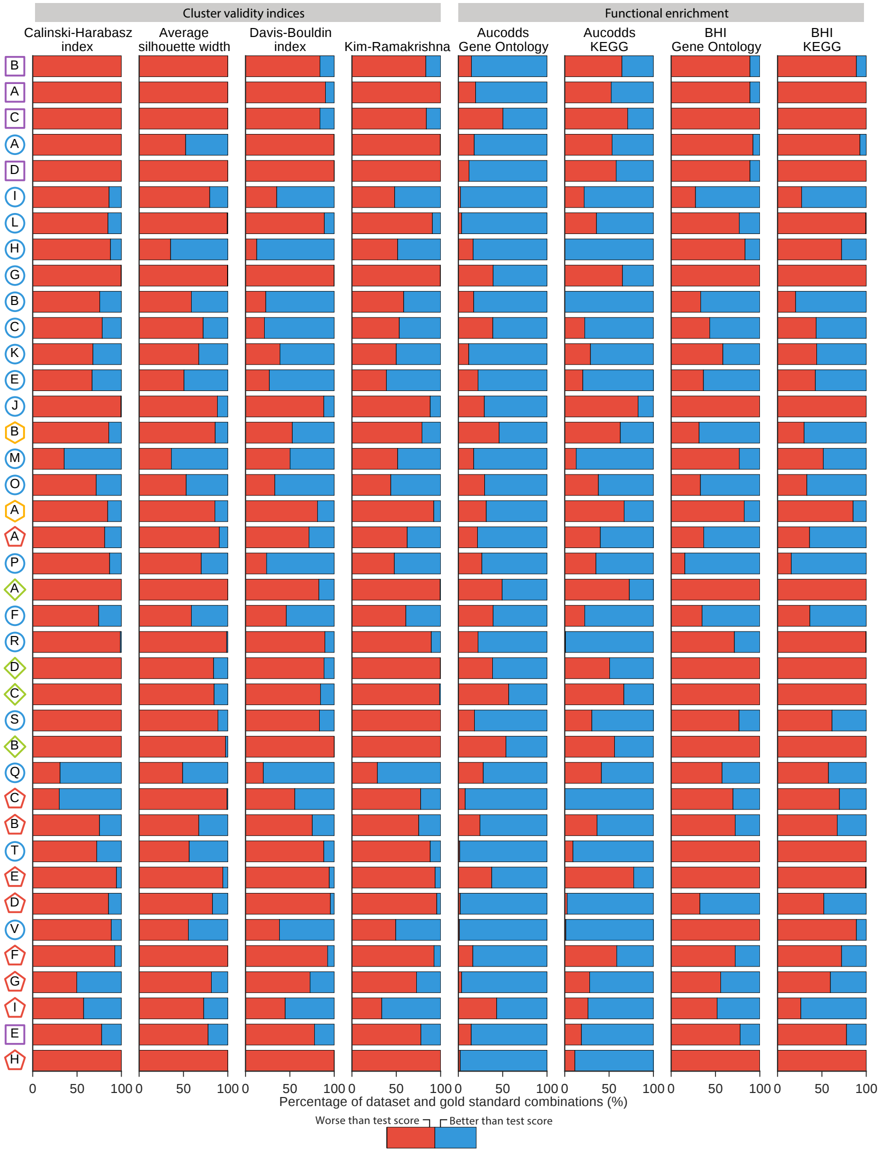

Supplementary Figure 10: Comparison between automatic parameter estimation methods and optimizing parameters on other datasets.
Shown are the percentage of dataset and gold standard (module definition and regulatory network) combinations where automatically estimating the number of parameters is better than using the most optimal parameters from another dataset (as given by the test score). This shows that even when a certain method is on average good at estimating the parameters of a particular method, none of the parameter estimation methods consistently perform well on every single combination of datasets and gold standards.

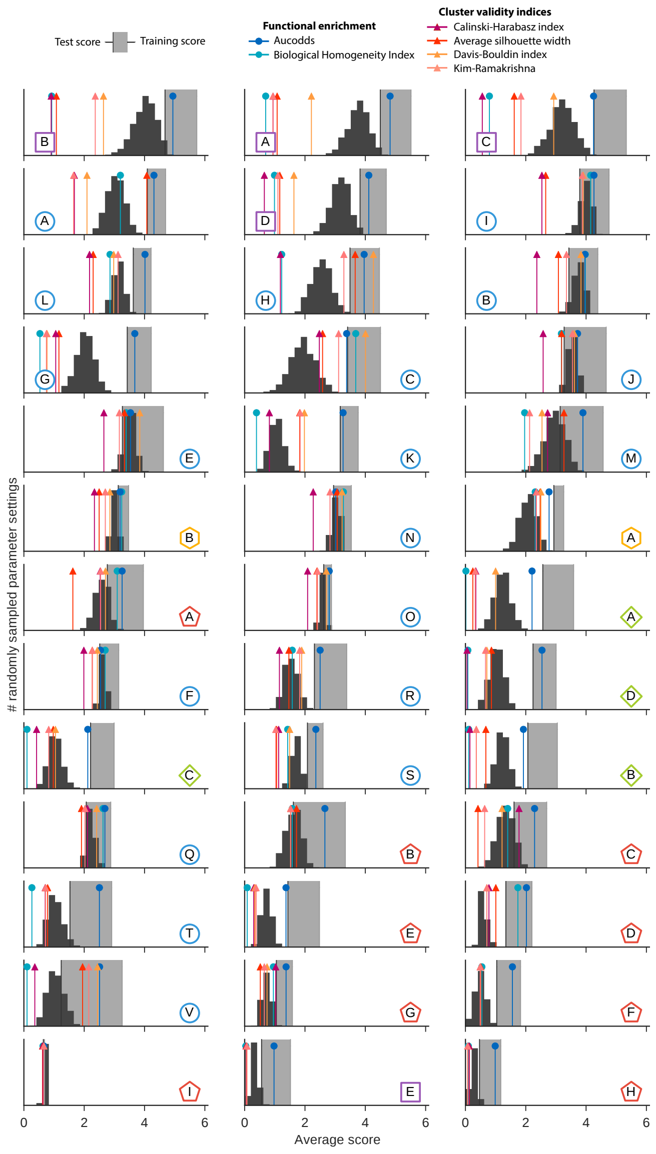

Supplementary Figure 11: Comparing the automatic estimation of parameters with randomly selecting parameters.
Shown in dark grey are the distribution of the score (x-axis) obtained after randomly selecting a set of parameters from those explored within the grid-search (**Supplementary Note 2**) across different datasets. Other annotations are similar as in **Figure 3**. The different markers and colors denote the score after automatically estimating parameters using either a cluster validity index or some measure looking at functional enrichment. The average training score (the most optimal score across all parameters) and average test scores (the score at those parameters which were optimal for another dataset) are shown as a grey background window. Current cluster validity indices usually only perform better than random when used with clustering methods, while measures based on functional enrichment (using the Gene Ontology database) usually work well across all different method categories.

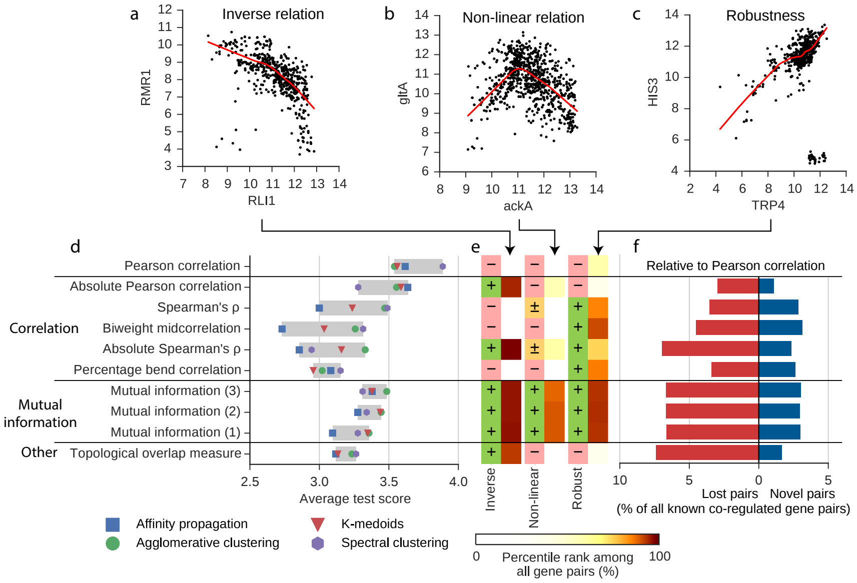

Supplementary Figure 12: Effect of alternative similarity metrics on the performance of clustering methods. One of the most important parameters for some clustering methods is the similarity or distance measure used to compare genes. The most popular measure, the Pearson correlation, assesses the extent towards which the expression of two genes is linearly correlated among all samples. Several alternative measures have been proposed (**Supplementary Note 3**), for handling inverse relationships, non-linear effects or improve the robustness of the measure. Here we evaluated these alternative measures on four of the top clustering methods which require a similarity or distance matrix as input. (**a**) Example of an inverse relation between two known co-regulated genes (RLI1 and RMR1) in the DREAM5 yeast dataset. (**b**) Example of a non-linear relation between two known co-regulated genes (gltA and ackA) in the DREAM5 *E. coli* dataset. (**c**) Example of a relation between two known co-regulated genes (TRP4 and HIS3) with a skewed distribution and outliers. (**d**) Performance of four clustering methods with different similarity measures, averaged over datasets and module definitions. (**e**) For every limitation of the Pearson correlation we assessed whether alternative measures can handle it theoretically (+,± and -). Can the metric handle inverse relations (+)? Can the metric detect non-linear monotonic relations (±) or more complex non-linear relations (+)? Can the method either handle outliers and/or skewed distributions (+)? Shown next to the theoretical properties are three case studies from **a**, **b** and **c**. Given are the rank percentages of every case study among all gene pairs in the datasets (higher is better). (**f**) Percentage of known co-regulated gene pairs removed (red) and gained (blue) between the Pearson correlation and an alternative metric within the top 10% of all gene pairs.

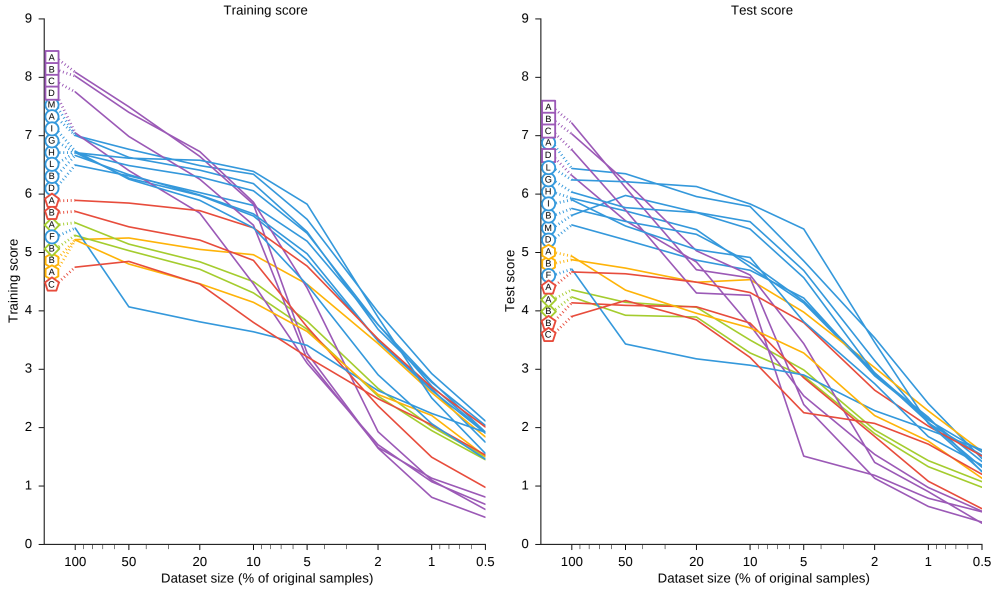

Supplementary Figure 13: Effect of a reduced number of samples on the performance of top module detection methods.
A subset of samples (x-axis) were randomly sampled, the performance of top methods (best methods within every module detection category) was again assessed using a grid search parameter exploration. We found that the performance of decomposition methods was more sensitive to a reduced number of samples, both for training and test scores.

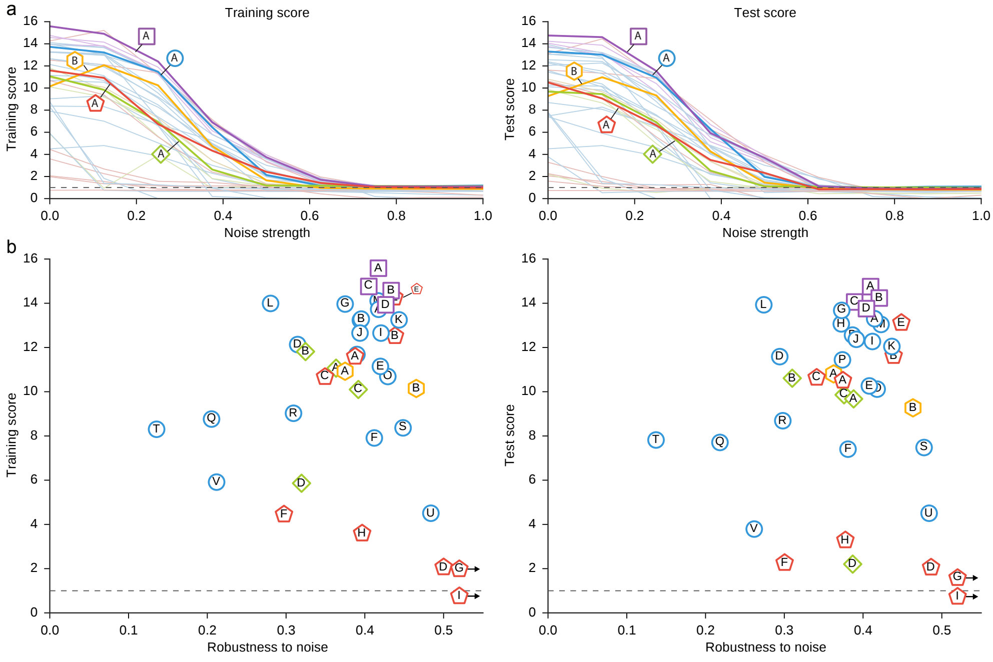

Supplementary Figure 14: Effect of noise on the performance of module detection methods
We used synthetic data to assess the influence of noise on the performance of the different methods. **(a)** Different levels of noise (noise strength, x-axis) were generated by changing the variance of normal and lognormal noise distributions within the GeneNetWeaver program. We found that most top methods had a comparable decrease in performance with increasing noise strength. Performance of other methods is shown in the background. **(b)** This was even more pronounced when comparing the robustness to noise (calculated by dividing the average performance among all noise strength levels over the initial performance without noise) with baseline performance, with some exceptions such as WGCNA (clustering method L).

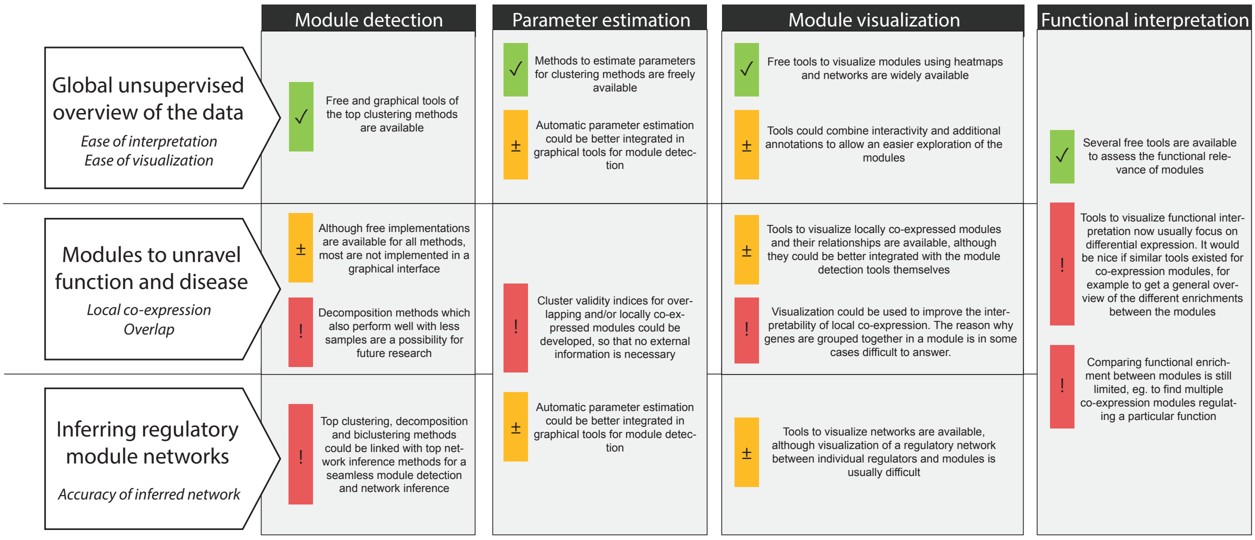

Supplementary Figure 15: Recommendations for future development for the detection and interpretation of modules in gene expression data.
Counterpart of **Figure 5** for developers of module detection methods. We list some aspects for developers of methods which have already been accomplished (green), primarily with regards to generating a global unsupervised overview of the data using clustering methods. In addition, we list some ongoing challenges, primarily with the parameter estimation, visualization and interpretation of biclustering and decomposition methods (orange and red).

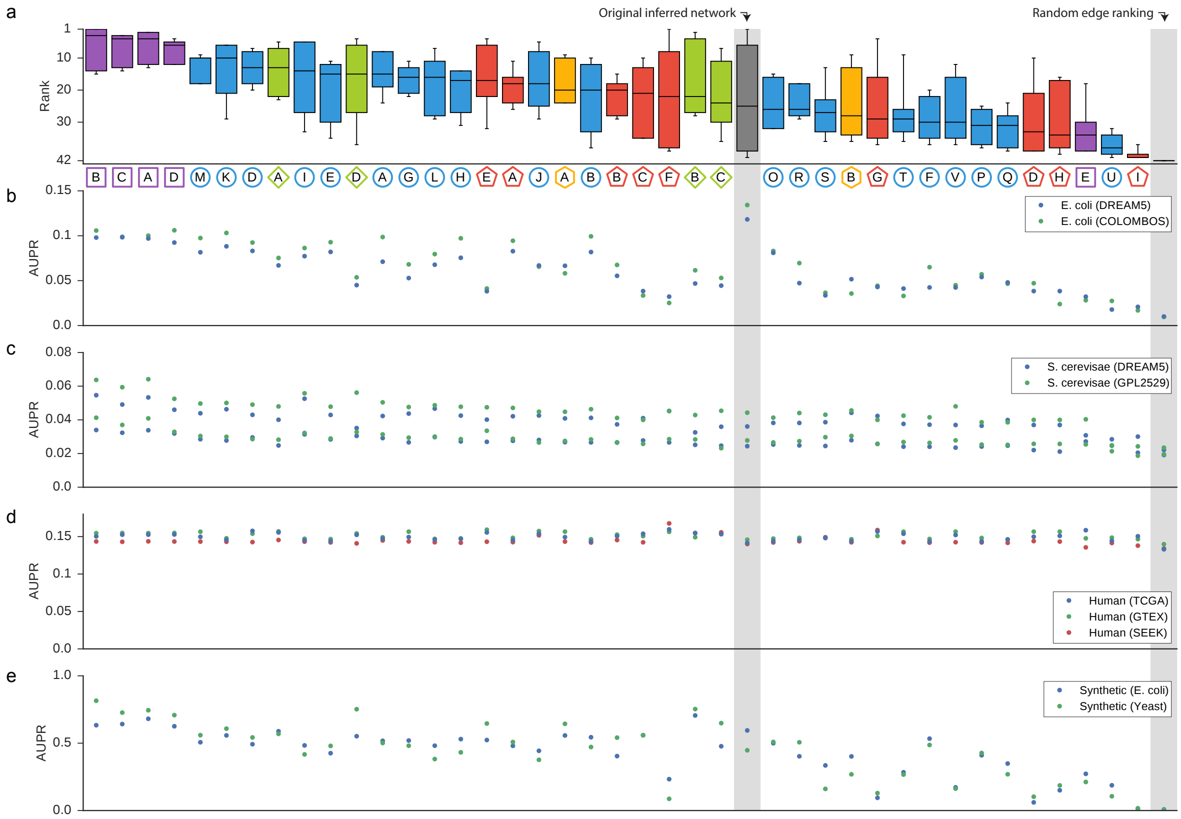

Supplementary Figure 16: Performance of the inferred network when combining modules from different module detection methods with direct network inference.
Modules can be used to improve the interpretability and the quality of an inferred regulatory network. Here we compared the accuracy of the inferred network when using a state-of-the-art direct network inference method (GENIE3) in combination with different module detection methods. Starting from the output of GENIE3 (a weighted network between regulators and target genes), we first calculated a weighted network between modules and regulators by averaging the weights of all genes within the module. From this, we again calculated a weighted network between regulators and individual target genes by determining for every regulator and target pair its maximal weight within the module network. The accuracy of this network was then assessed using the standard area under the precision-recall curve metric (AUPR). We found that the modules from decomposition methods generally lead to the most accurate inferred network. However, the advantage of including modules to improve the inferred network was only clear on yeast and synthetic data, as the performance slightly decreased on *E. coli* data and the increase of performance on human data was negligible.

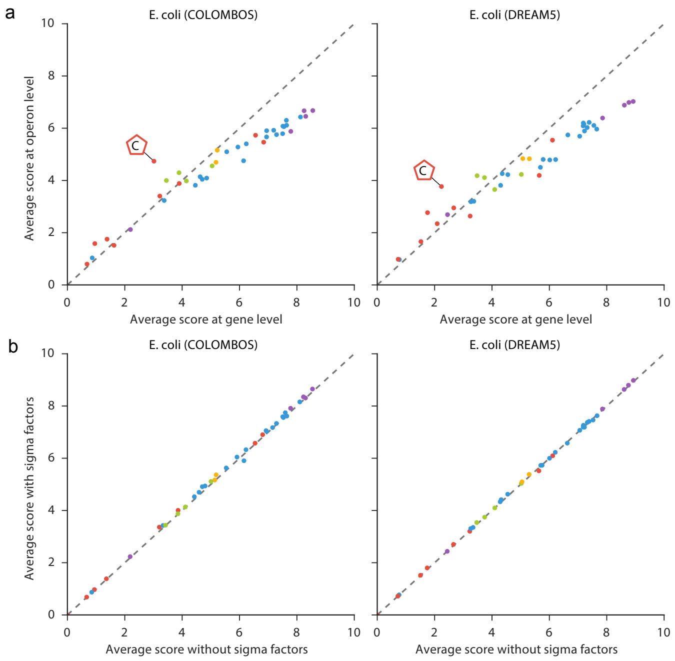

Supplementary Figure 17: Effect of working on the operon level or adding sigma factor interactions. **(a)** Genes within an operon typically have similar expression values due to co-transcription. We assessed whether merging the expression profiles of the genes within an operon, together with their regulatory interactions, would have an effect on the relative performance of the methods. We found that while the performance was slightly lower for most methods when merging the operons, the overall ranking of the methods was not severely affected. **(b)** We also found that including regulatory links between sigma factors (excluding the basal sigma factor) and target genes has a negligible effect on the performance of the methods.

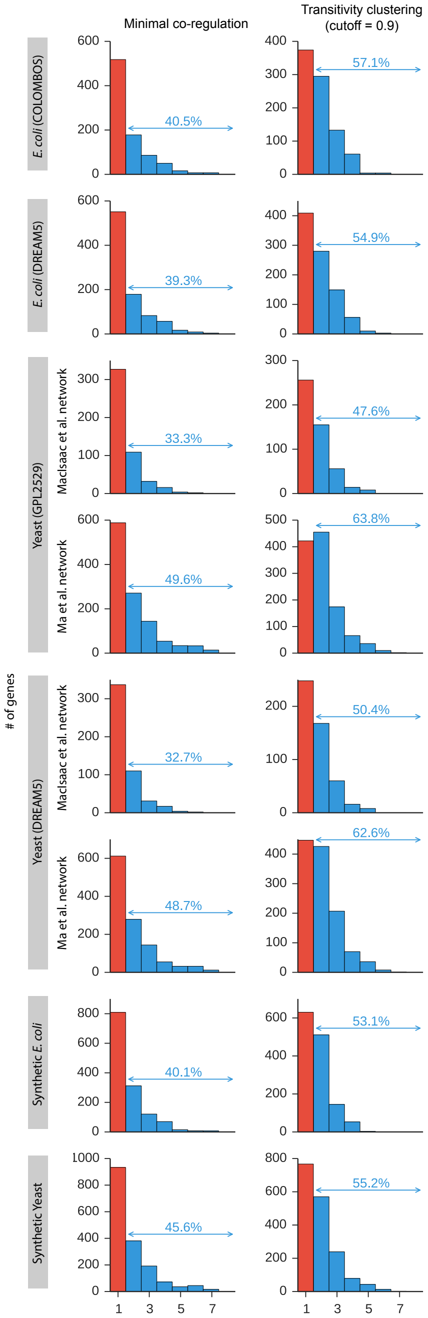

Supplementary Figure 18: Membership distributions for two module definitions in which overlap was prevalent.
These distributions show the number of genes (y-axis) which are part of one (red) or more (blue, x-axis) modules. We found that irregardless of the network a similar number of genes was part of more than one module, 30%-40% in the case of minimal co-regulation and 50%-60% in the case of a particular interconnected subgraph definition (transitivity clustering with $cutoff = 0.9$). The presence of overlap in the gold standard could allow methods which can detect overlapping modules (such as most biclustering and decomposition methods) to outperform other methods which can not handle overlap (such as most clustering methods).

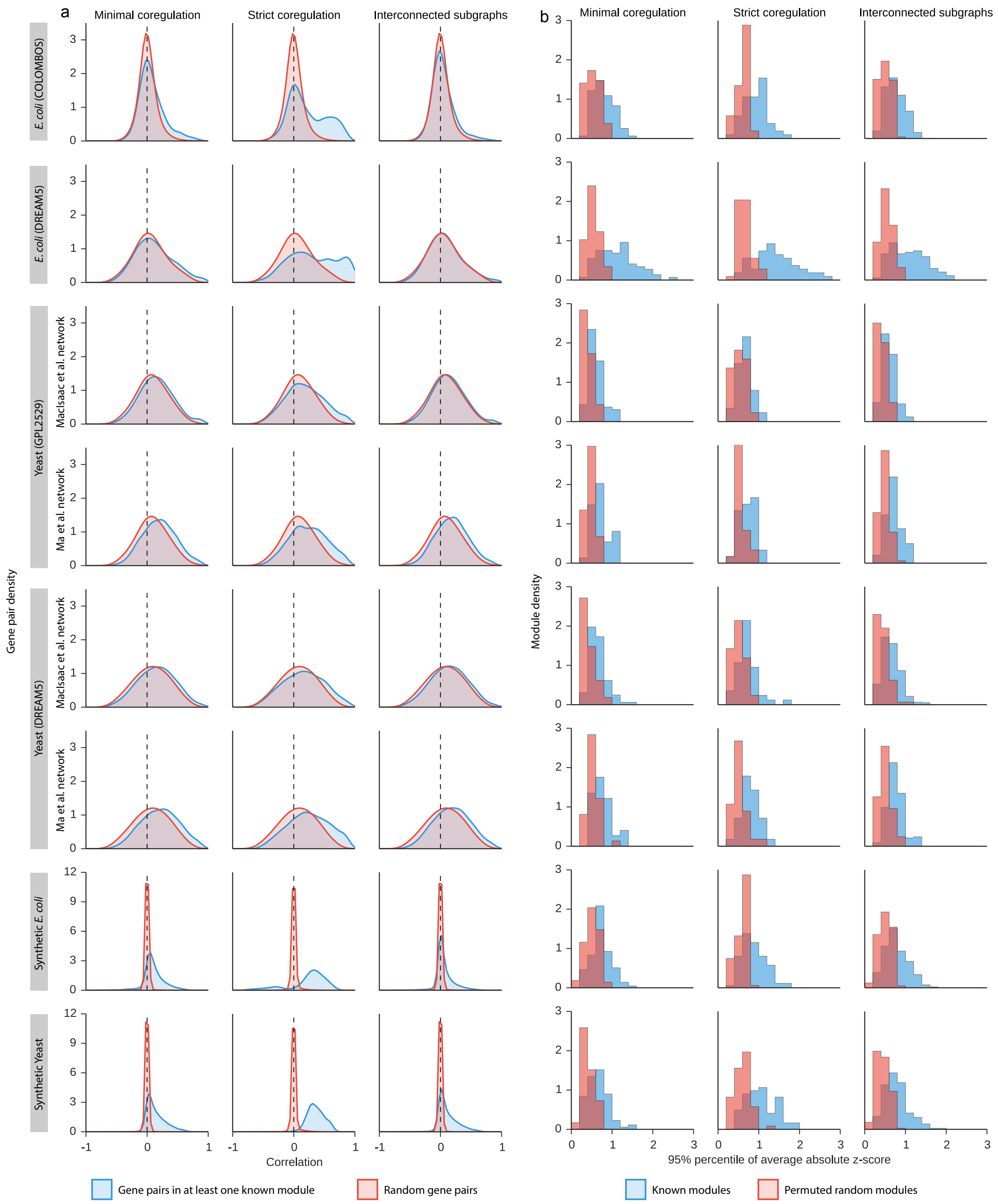

Supplementary Figure 19: Co-expression of the known modules.
To determine whether the expression datasets contain the known modules, we assessed whether the known modules are globally or locally co-expressed. **(a)** Distributions of the correlation (x-axis) between gene pairs within at least one known modules (according to three module definitions, where the distributions of the interconnected subgraph definitions were merged), compared with random gene pairs. Gene pairs within a known module are more frequently positively co-expressed compared with random gene pairs, especially on *E. coli* and synthetic datasets. (**b**) Local co-expression of the known modules, compared with randomly permuted modules, based on the extreme biclustering definition employed by biclustering methods such as ISA and QUBIC. Extreme biclusters are defined as groups of genes which are relatively highly (or lowly) expressed in (at least) a subset of samples. We assessed this for every module by first transforming the expression matrix to z-scores ($\mu = 0$ and $\sigma = 1$ for every gene). If a module is locally co-expressed in 5% of the samples, it will (according to the extreme bicluster definition) have a high average absolute z-score in 5% of the samples. We therefore show here the distributions of the 95% percentile of these average absolute z-scores across different modules. This figure indicates that the known modules are also more locally co-expressed compared with randomly permuted modules.

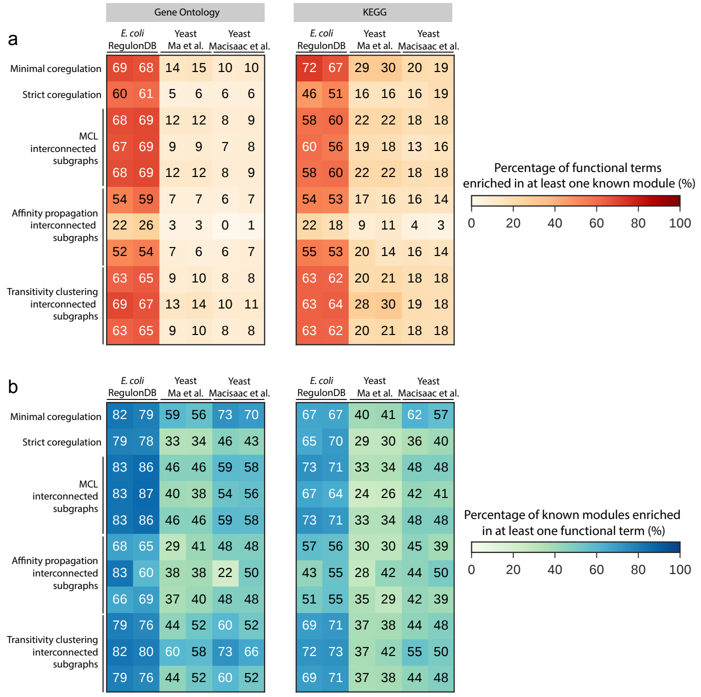

Supplementary Figure 20: Functional enrichment of known modules.
(**a**) The functional coverage of the known modules, i.e. how well do all known modules cover the known functional space, given by non-overlapping Gene Ontology terms and KEGG pathways. This is much higher on *E. coli* datasets compared to yeast, indicating that the regulatory networks of *E. coli* are relatively more complete compared with those of yeast. (**b**) Percentage of known modules enriched in at least one functional term. In *E. coli* a large majority of known modules are enriched, while on yeast data this value varies around 50%.
Together, this indicates that the known modules on *E. coli* have relatively better quality, one possible explanation for why the performance on *E. coli* data is generally higher than on yeast data.

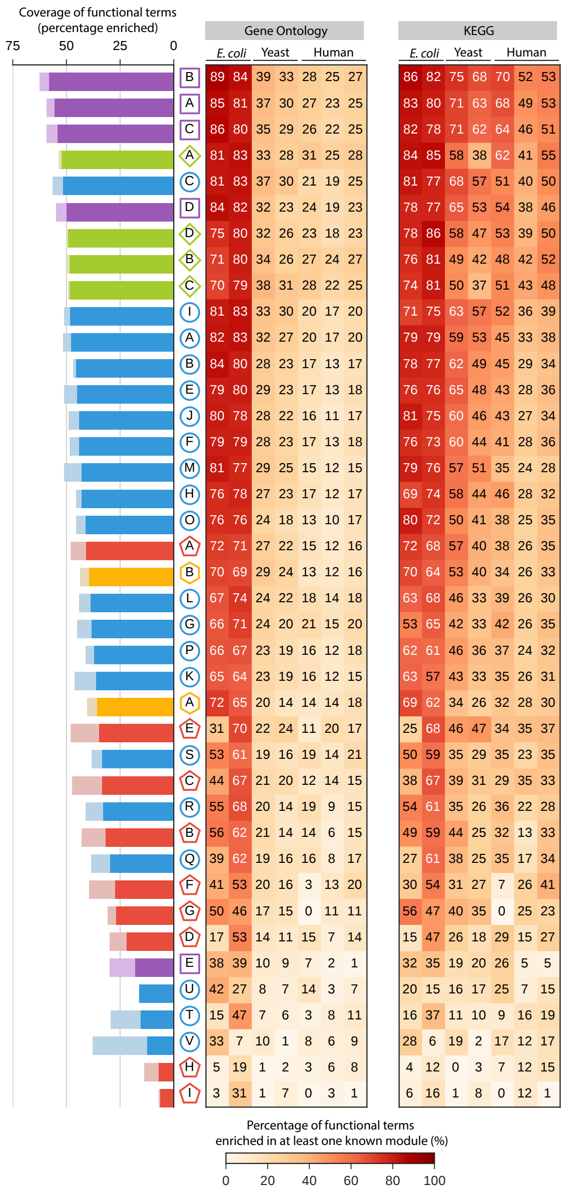

Supplementary Figure 21: Functional coverage of observed modules.
We assessed how well the observed modules of different methods cover the (non-overlapping) functional space of Gene Ontology terms and KEGG pathways. **(a)** Average coverage across *E. coli*, yeast and human datasets. We calculated both the average coverage when using the most optimal parameters (light color) or the average coverage at the most optimal parameters of another dataset (dark color). Decomposition and direct NI methods have the best coverage of functional space, followed by clustering and iterative NI methods. **(b)** Average functional coverage (test scores) on individual datasets. The observed modules generally cover a larger part of the functional space on *E. coli* data compared with yeast and human, although for yeast data the coverage is still considerably larger compared with the known modules (**Supplementary Figure 20**).

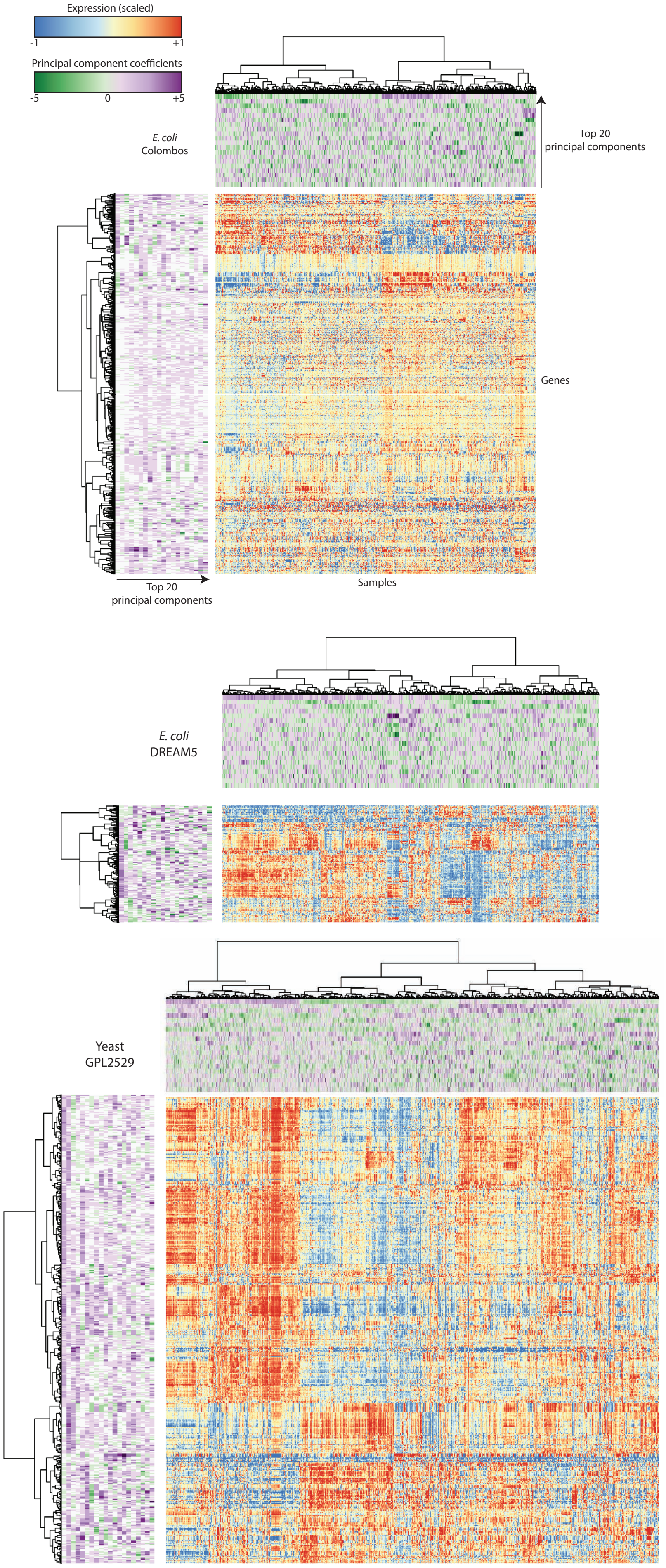

Expression (scaled)
-1
+1
Principal component coefficients
-5
0
+5
E. coli
Colombos
Top 20
principal components
Genes
Top 20
principal components
Samples
E. coli
DREAM5
Yeast
GPL2529

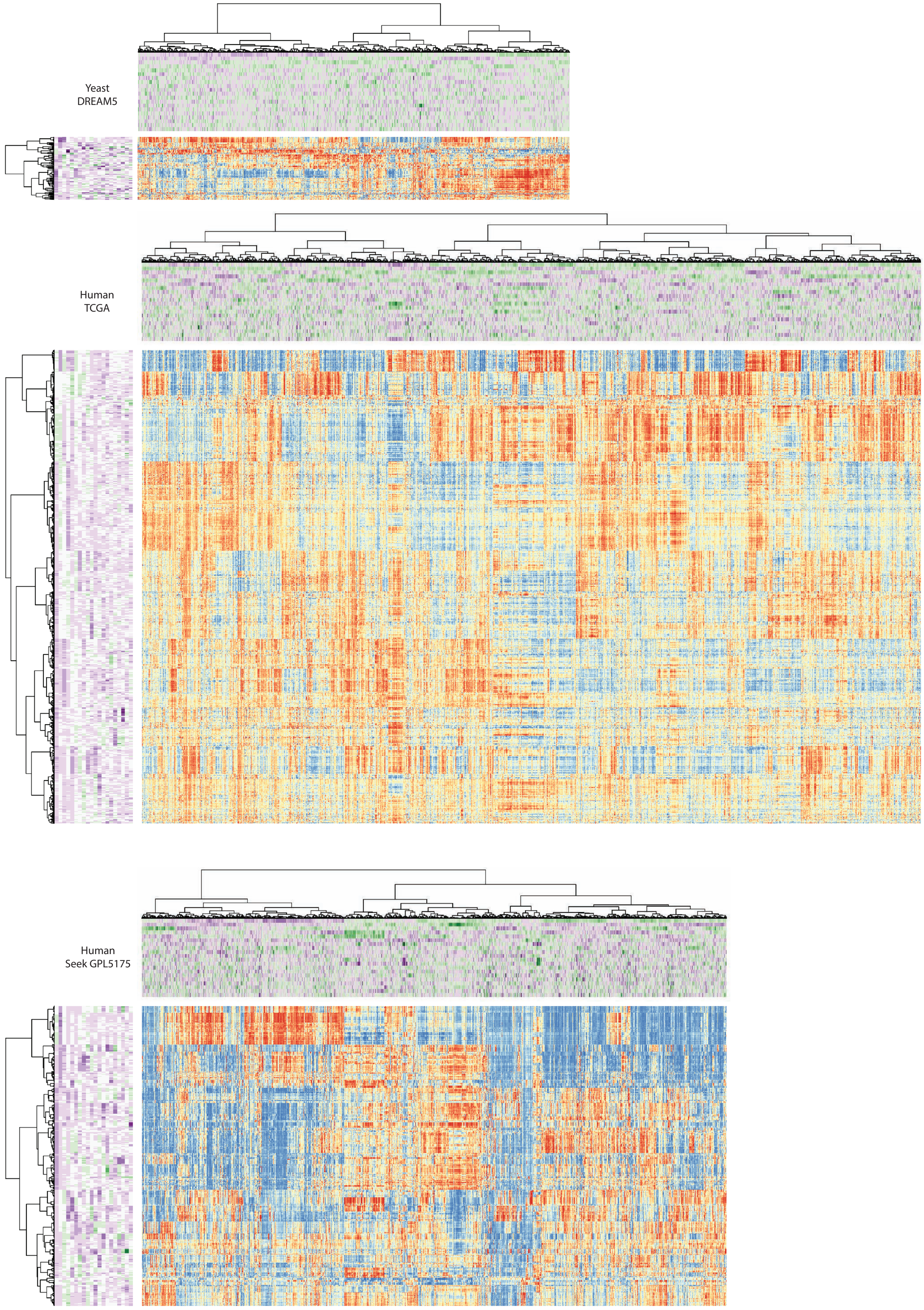
Yeast
DREAM5
Human
TCGA
Human
Seek GPL5175

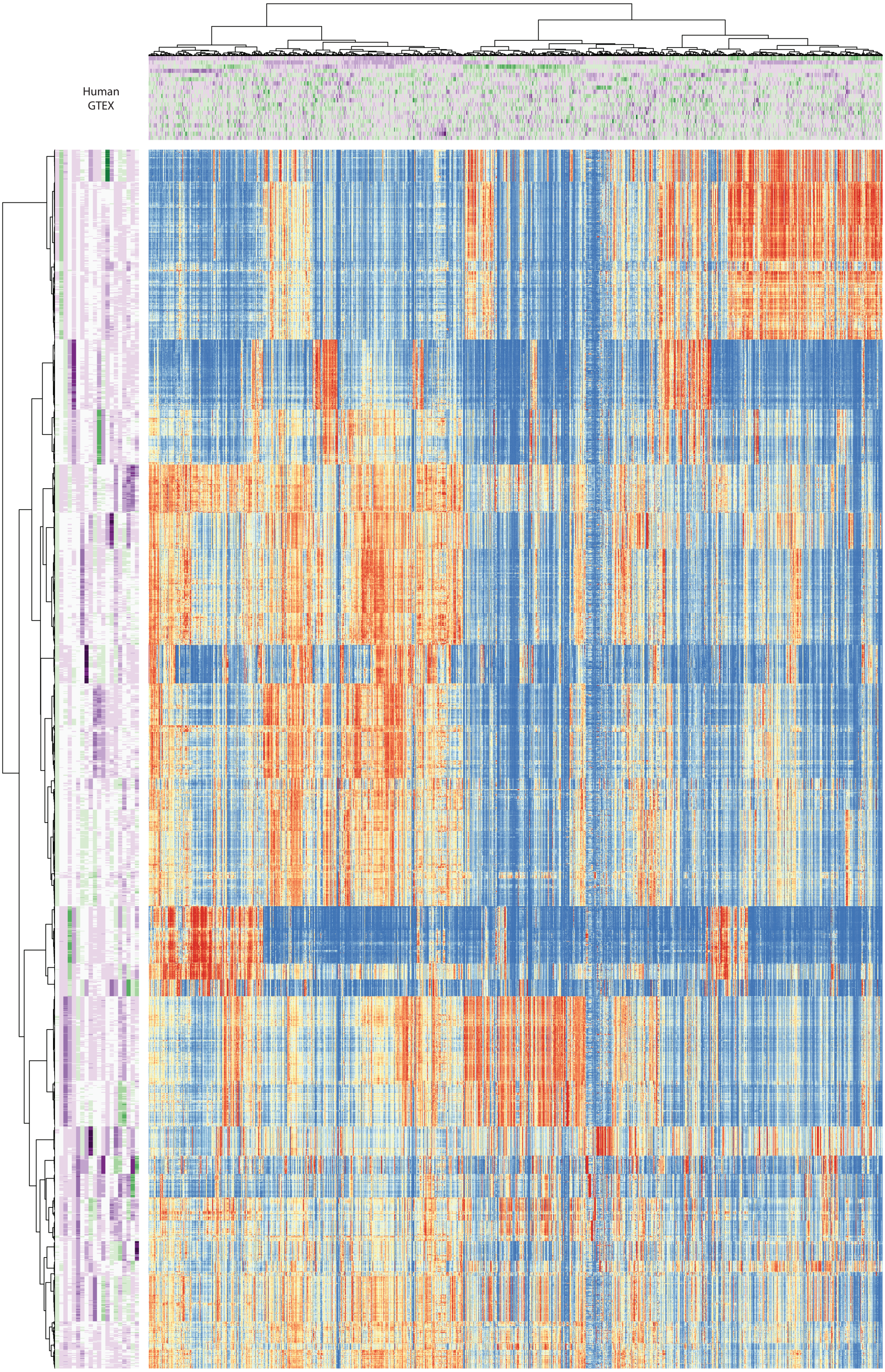

Supplementary Figure 21: Heatmaps of the expression datasets used in this study.
Shown are expression values (red - orange - blue) and the first 20 principal components (purple - white - green) for both the gene (rows) and sample (columns) dimensions. Dendrograms were created through hierarchical clustering using Ward's criterion.

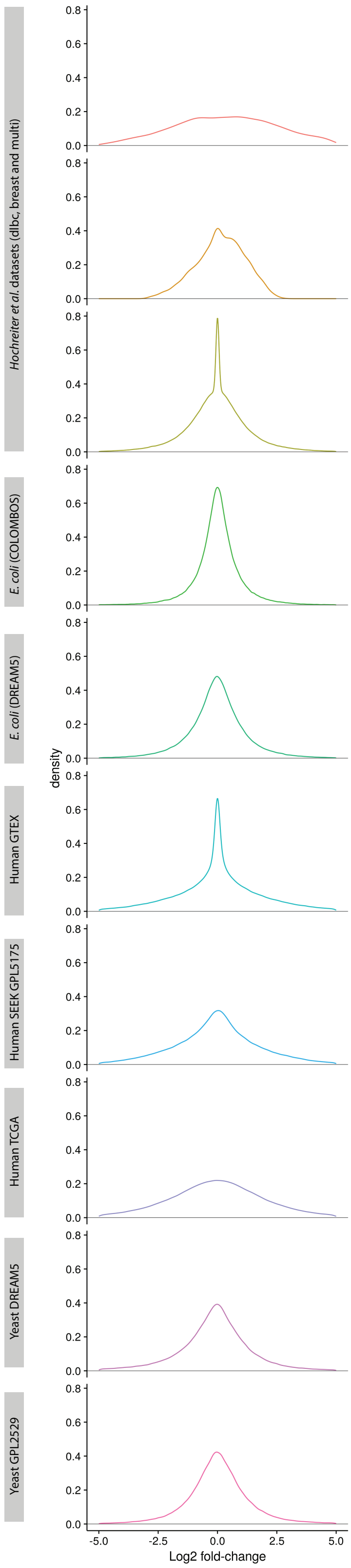

Supplementary Figure 22: Distribution of log-fold changes between all genes and samples, for three datasets analyzed by Hochreiter et al. [38] and datasets used in this study. Some datasets contain large changes in gene expression (with high fold-changes) while others contain more subtle changes.

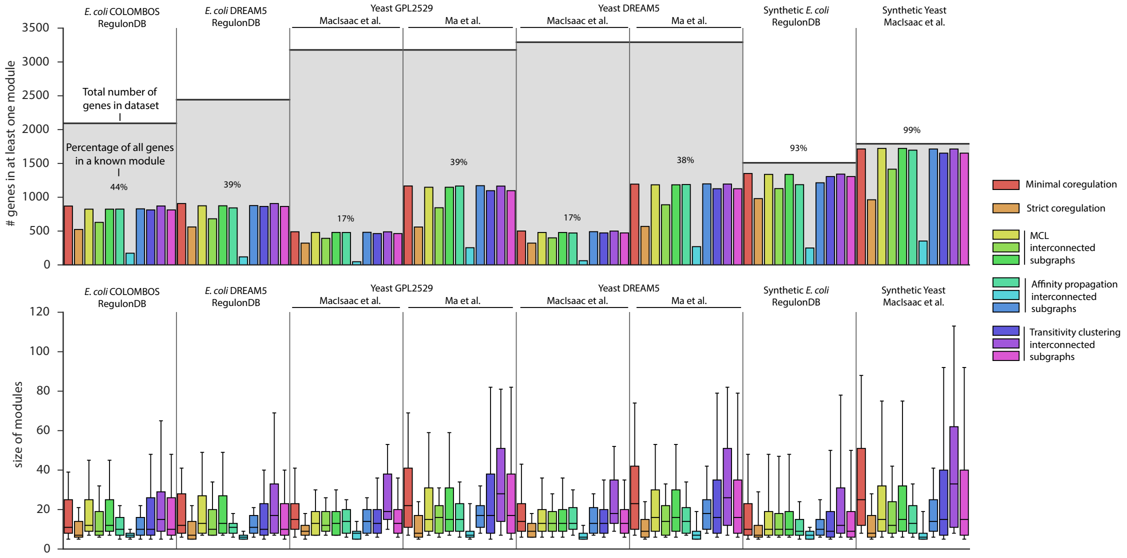

Supplementary Figure 23: Characterization of the known modules with respect to the coverage of all genes (top) and the size (bottom).

Whiskers denote 10% and 90% weighted percentiles, while the box denotes 25% and 75% percentiles.

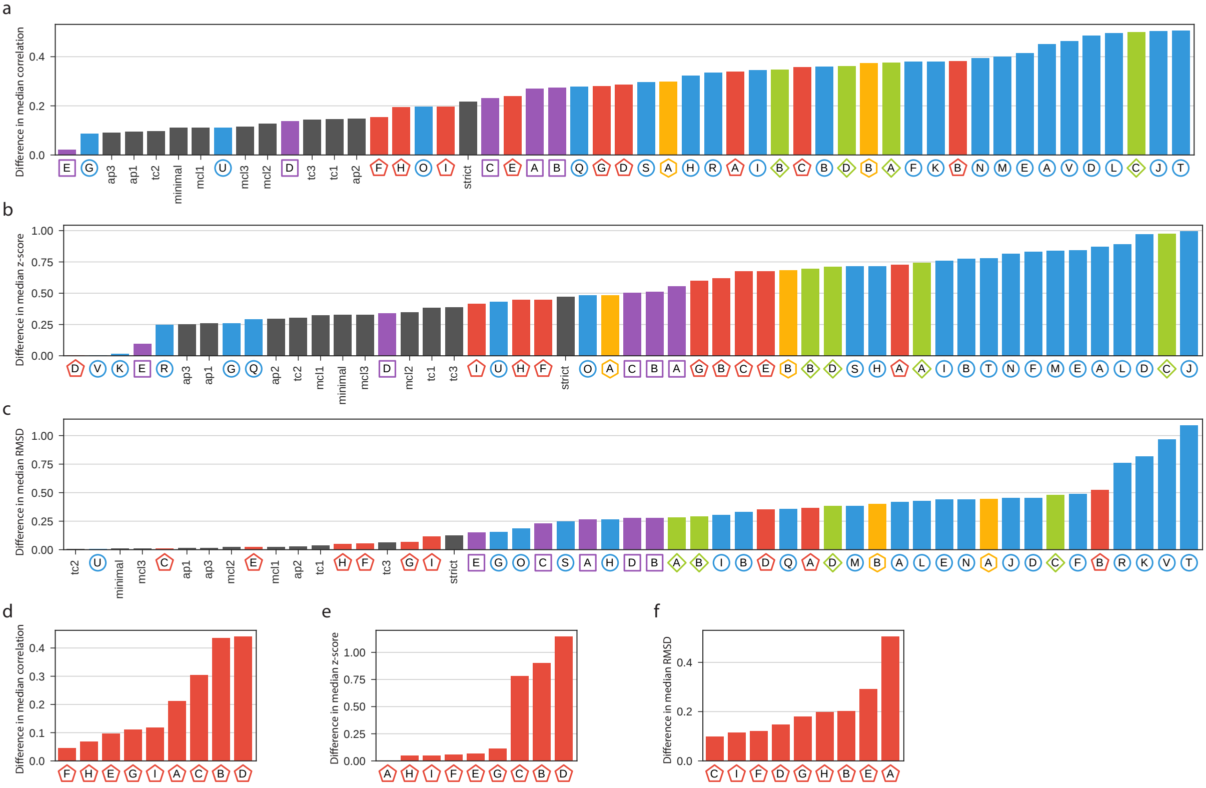

Supplementary Figure 24: Co-expression of modules detected by module detection methods and known modules.

Three co-expression measures were calculated, based on the three main bicluster types. To calculate the strength of co-expression, we calculated the median of the difference between the co-expression of a true module and its permuted version. **(a)** Co-expression based on the average correlation between gene pairs within a module, measuring how well the expression profiles are similar within a module. **(b)** Co-expression based on the average top 5% z-score, measuring how extreme the expression is within a module. **(c)**: Co-expression based on the root mean squared deviation within each module, measuring how constant the expression is within a module. **(d-f)** Similar as **a b** and **c** but looking at co-expression within the biological samples of a bicluster.

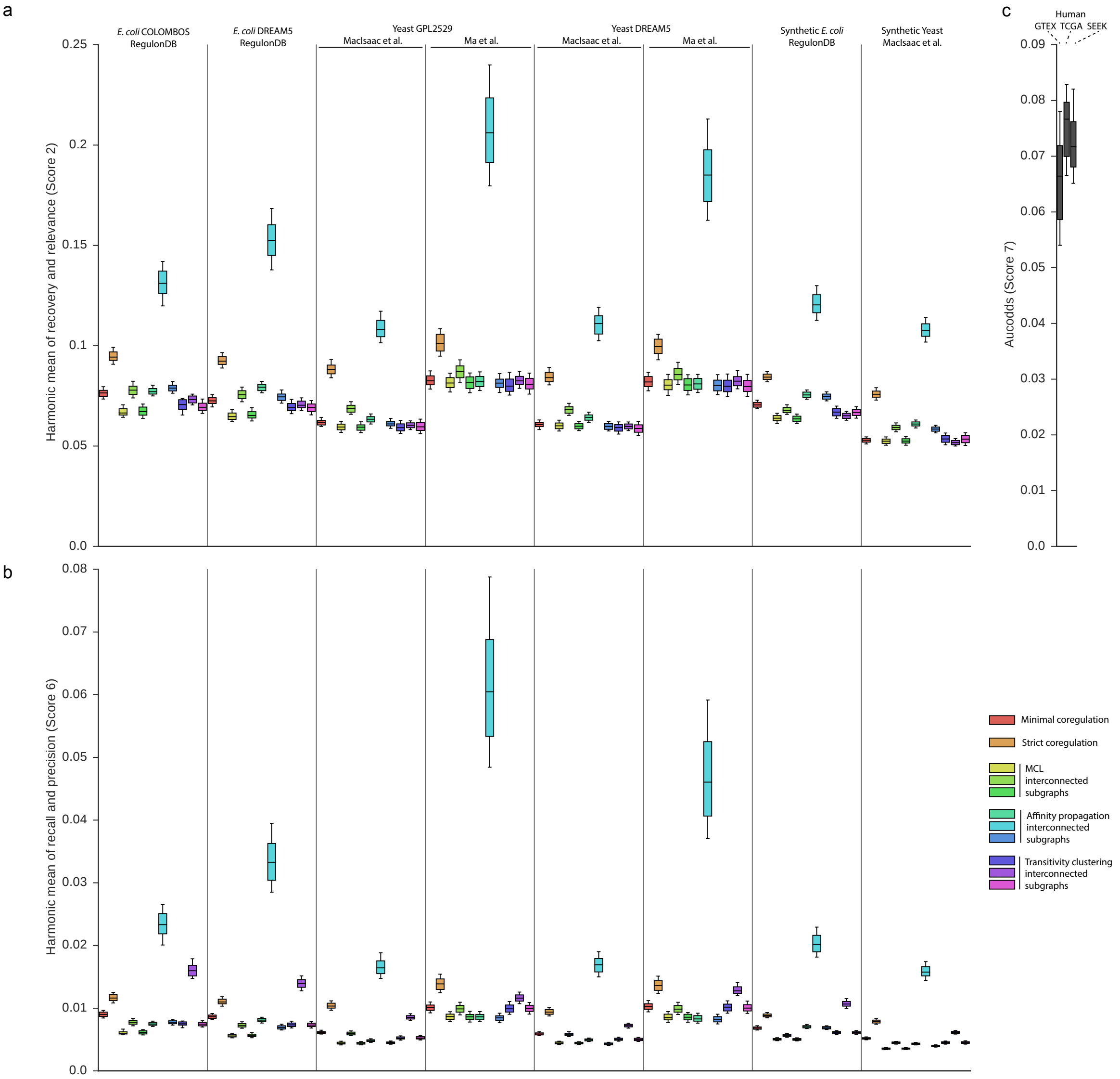

Supplementary Figure 25: Variability of scores of permuted modules
Shown are distributions of the scores when using randomly permuted known modules to assess performance ($n = 500$). Whiskers denote 10% and 90% weighted percentiles, while the box denotes 25% and 75% percentiles.

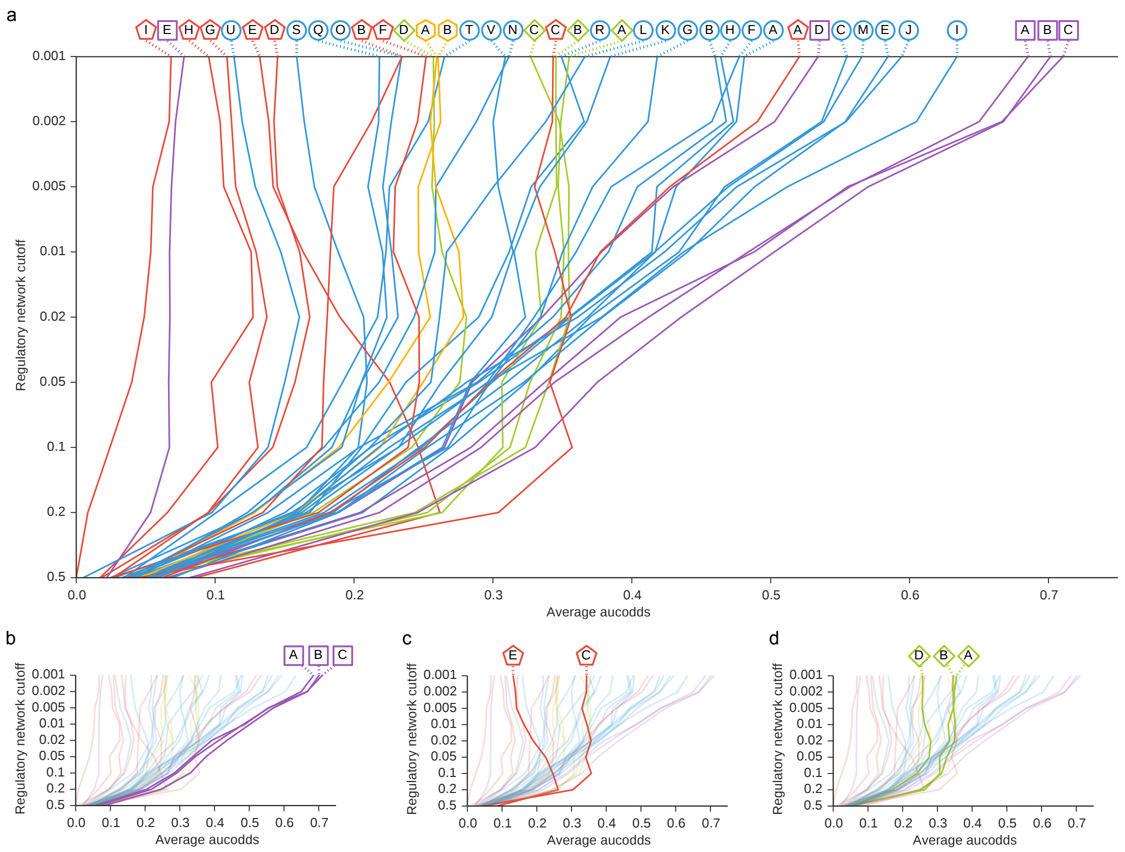

Supplementary Figure 26: Effect of network cutoff on human datasets. Average aucodds scores across the three different human datasets at different cutoff values of the gold standard regulatory network. Generally, the performance of methods decreases with increasing stringency (**a, b**), although the performance of some biclustering methods (**c**) and direct NI methods (**d**) remains stable for much longer.

Supplementary Note 1: Measures for comparing overlapping modules

Numerous scores have been proposed to compare clusterings of data [55–58], but most of these scores have problems with handling overlap[1] and/or non-exhaustive cluster assignment[2], which has already been discussed elsewhere [58] and which we here further illustrate using 12 small test cases (**Supplementary Note 1 Figure 1**). We define two different sets of known modules, without overlap (1-6) and with overlap (7-12). A perfect match between the observed modules and known modules is given in case 1 and 7. In every other test case the observed modules do not perfectly correspond with the known modules, and therefore the score of these test cases should become worse compared to case 1 or 7. However, as shown in **Supplementary Note 1 Table 1**, none of the classical clustering scoring metrics fulfill this criterion. Most scores have issues when the known modules and/or observed modules overlap with each other, as the performance on cases 4-6 and 11-12 stays the same or even increases compared to the perfect case. Only one score can perfectly handle overlap (F-measure [56]), but it has problems handling non-exhaustive cluster assignment, as evidenced by its perfect score on cases 2 and 9.

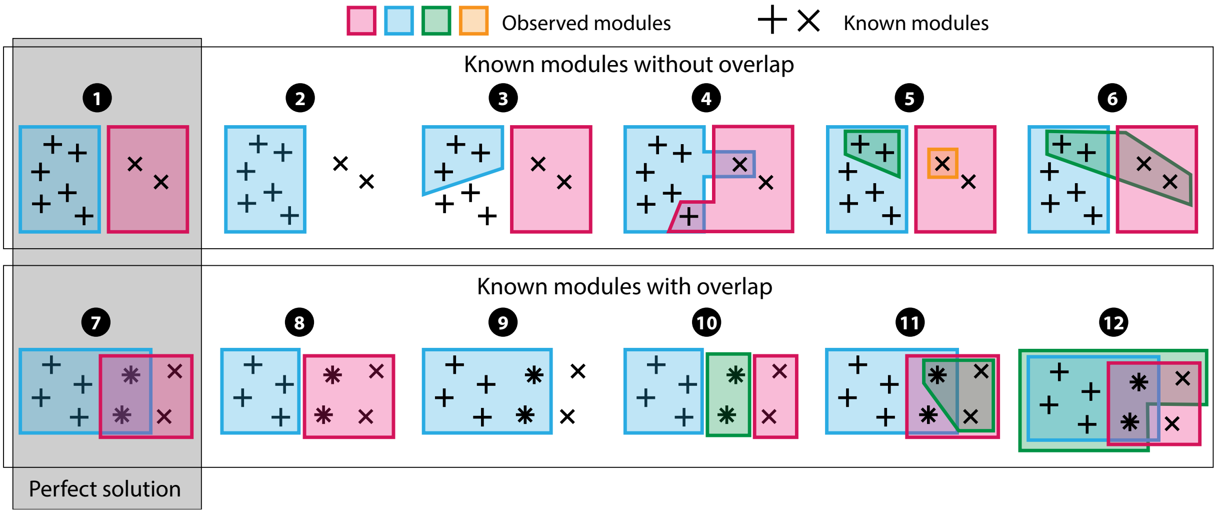

Supplementary Note 1 Figure 1: 12 test cases to assess scores for comparing two sets of potentially overlapping modules.

Several alternative scores have been proposed in literature to better handle potential overlap between clusters/modules. In the following formulas, we use these conventions: G represent all genes, M a set of known modules, M' a set of observed modules, $M(g)$ the modules which contain g and $E(g, M)$ the set of genes which are together with g in at least one module of M (including g itself).

One family of measures, which includes the recovery and relevance scores used in this study, have already been extensively applied within the biclustering literature [44, 59]. Similar scores have also independently been described elsewhere [60, 61]. These scores are calculated in two steps. First a similarity/distance matrix is calculated between the two sets of modules. There are several possibilities for this similarity score, such as the Jaccard index [59] or entropy based measures [60]. In the next step the similarity values are summarized in one number by mapping known modules to observed modules and vice versa. A score quantifying the false positives (S_1) is calculated by summing/averaging the similarities for every observed modules by selecting the best representative in the known modules. Similarly, a score quantifying the false negatives (S_2) is calculated by summing/averaging the similarities for every known modules by selecting the best representative in the observed modules. These two scores can then be combined in a final score giving the trade-off between false positives and false negatives by summing or averaging S_1 and S_2.

Turner et al. [62] used an asymmetric measure for module similarity:

$$S_1 = \text{Sensitivity} = \frac{1}{|M'|} \sum_{m' \in M'} \max_{m \in M} \frac{|m' \cap m|}{|m|}$$

$$S_2 = \text{Precision} = \frac{1}{|M|} \sum_{m \in M} \max_{m' \in M'} \frac{|m' \cap m|}{|m'|}$$

$$S = \frac{2}{\frac{1}{S_1} + \frac{1}{S_2}} \qquad \textbf{(Score 1)}$$

[1]Defined as at least one gene belonging to multiple modules
[2]Defined as certain genes not included in any modules

	Known modules without overlap						Known modules with overlap					
	1	2	3	4	5	6	7	8	9	10	11	12
F-measure [55]	1.00	0.75	0.50	1.11	1.21	1.33	1.25	0.90	0.75	0.73	1.57	2.06
F-measure [56]	1.00	1.00	0.77	0.89	0.81	0.77	1.00	0.89	1.00	0.67	0.96	0.97
1-FDR [56]	1.00	0.94	0.25	1.00	1.00	1.00	1.00	0.60	0.75	0.40	1.00	1.00
1-FPR [56]	1.00	0.92	0.50	1.00	1.00	1.00	1.00	0.50	0.62	0.40	1.00	1.00
FMI [56]	1.00	0.97	0.50	0.83	1.00	0.89	1.00	0.77	0.87	0.63	1.00	0.91
Jaccard [56]	1.00	0.94	0.25	0.70	1.00	0.80	1.00	0.60	0.75	0.40	1.00	0.83
Rand [56]	1.00	0.96	0.57	0.75	1.00	0.86	1.00	0.71	0.82	0.57	1.00	0.86
Sensitivity [56]	1.00	1.00	1.00	0.70	1.00	0.80	1.00	1.00	1.00	1.00	1.00	0.83
Specificity [56]	1.00	0.92	0.50	1.00	1.00	1.00	1.00	0.50	0.62	0.40	1.00	1.00
V-measure [56]	1.00	0.00	1.00	0.22	0.67	0.49	0.13	0.43	0.00	0.46	0.12	0.07
Purity [57]	1.00	0.75	0.62	1.00	1.38	1.25	1.25	1.00	0.75	1.00	1.62	2.00
Entropy [57]	0.56	0.00	0.67	0.61	1.17	1.01	0.68	0.67	0.00	1.05	1.06	1.09

Supplementary Note 1 Table 1: Comparison of different measures for comparing two sets of modules, based on the test cases described in Supplementary Note 1 Figure 1.
These metrics are frequently used to compare different non-overlapping and exhaustive clusterings. Compared with the score on test cases 1 and 7 (grey), a good measure should consequently score lower on cases 2-6 and 8-12 (green). This condition is not satisfied by any of the measures.

The "Precision" score was originally named the "Specificity" in this study, but the actual meaning relates more closely to the common usage of precision as it estimates how well the observed modules are also known.

Prelić et al. [59] used a symmetric measure for module similarity, the Jaccard index:

$$\begin{aligned} S_1 = \text{Recovery} &= \frac{1}{|M|}\sum_{m\in M}\max_{m'\in M'}\text{Jaccard}(m',m) \\ S_2 = \text{Relevance} &= \frac{1}{|M'|}\sum_{m'\in M'}\max_{m\in M}\text{Jaccard}(m',m) \\ \text{Jaccard}(m',m) &= \frac{|m'\cap m|}{|m'\cup m|} \\ S &= \frac{2}{\frac{1}{S_1}+\frac{1}{S_2}} \end{aligned} \quad \textbf{(Score 2)}$$

Hochreiter et al. [38] proposed a slightly modified version of the Recovery and Relevance. They added an additional constraint so that every known module can only be mapped to one observed module and vice versa. If $p_i = \{m_i, m_i'\}$ represents a pair of a known module m and observed modules m', the consensus score is defined as

$$\text{Consensus} = \frac{1}{\max(|M|,|M'|)}\sum_{p_i\in P}\text{Jaccard}(m',m) \quad \textbf{(Score 3)}$$

The known modules and observed modules are matched with each other so that the consensus score is maximized using the Hungarian algorithm (**Score 3**).

Goldberg et al. [61] proposed the Best Match scores, using the edit distance, jaccard index and an entropy based measure (based on earlier work by [60]) as similarity measures.

$$\begin{aligned} S_1 &= \frac{1}{|M|}\sum_{m\in M}\max_{m'\in M'}\text{Jaccard}(m',m) \\ S_2 &= \frac{1}{|M'|}\sum_{m'\in M'}\max_{m\in M}\text{Jaccard}(m',m) \\ S &= S_1 + S_2 \end{aligned} \quad \textbf{(Score 4)}$$

$$
\begin{aligned}
S_1 =& \frac{1}{|M|} \sum_{m \in M} \max_{m' \in M'} H(m'|m) \\
S_2 =& \frac{1}{|M'|} \sum_{m' \in M'} \max_{m \in M} H(m|m') \\
S =& S_1 + S_2
\end{aligned} \qquad \textbf{(Score 5)}
$$

Although **Score 2** and **Score 4** have the same S_1 and S_2, they differ in the way these two scores are aggregated, ie. a harmonic mean (**Score 2**) and a summation (**Score 4**). We did not consider the other scores proposed by Goldberg et al. [61] because they require one or more parameters and would add another source of potential bias in the analysis.

Another family of measures is based on the BCubed measure. First proposed in [63] to compare non-overlapping clustering, Amigó et al. [58] extended this measure to also handle overlap. Rosales-Méndez and Ramírez-Cruz [64] adapted the metric to make sure it can only reach the optimal value of 1 when the observed modules are the same as the known modules:

$$
\begin{aligned}
S_1 = \text{Recall} =& \frac{1}{|G|} \sum_{g \in G} \frac{1}{|E(g,M)|} \sum_{g' \in E(g,M)} \frac{\min(|M'(g) \cap M'(g')|, |M(g) \cap M(g')|) \cdot \Phi(g,g')}{|M(g) \cap M(g')|} \\
\Phi(g,g') =& \frac{1}{|M'(g,g')|} \sum_{m' \in M'(g,g')} \max_{m \in M(g,g')} \text{Jaccard}(m',m)
\end{aligned}
$$

$$
\begin{aligned}
S_2 = \text{Precision} =& \frac{1}{|G|} \sum_{g \in G} \frac{1}{|E(g,M')|} \sum_{g' \in E(g,M')} \frac{\min(|M'(g) \cap M'(g')|, |M(g) \cap M(g')|) \cdot \Phi(g,g')}{|M'(g) \cap M'(g')|} \\
\Phi(g,g') =& \frac{1}{|M(g,g')|} \sum_{m \in M(g,g')} \max_{m' \in M'(g,g')} \text{Jaccard}(m',m)
\end{aligned}
$$

$$
S = \frac{2}{\frac{1}{S_1} + \frac{1}{S_2}} \qquad \textbf{(Score 6)}
$$

While we also considered including "module preservation statistics" as proposed by Langfelder and colleagues [65], we found that these measures are primarily useful to assess whether individual modules are preserved within a network, but not whether all (or most) modules present within a network are found by a particular module detection method.

The structure and size of modules detected by module detection methods can vary wildly between methods and parameter settings. For instance, some parameter settings of decomposition methods will only assign a small number of genes to any module. Other parameter settings will assign all genes multiple times to several large modules. A good score should be robust against such extreme cases as they could be produced by certain methods during the parameter optimization procedure. We tested this based on an empirical experiment where we used a set of known modules (from the *E. coli* COLOMBOS dataset using the minimal co-regulation module definition) and compared them with several extreme cases of observed modules (**Supplementary Note 1 Figure 2**):

- Two trivial clustering examples. Putting all genes together in one large module had bad performance for all scores except for Score 5. Putting all genes in their own separate module resulted in bad performance for all scores except for Score 4.

- Permutations of the known modules. A certain percentage of all genes is mapped to a permuted version of these genes, and all instances of a gene within the known modules are replaced by the mapped version. As expected, in all cases permuting the known modules had severe effects on performance.

- Effect of using only a subset of all known modules. Again, performance decreased consistently between all scores.

- Effect of randomly adding extra genes to the known modules. Score 5 responded very strongly to this relative to other perturbations.

- Effect of randomly removing genes from known modules. Again, performance decreased in all scores, although the effect was relatively weak for Score 5.

- Randomly sampling modules from the full solution set (all possible modules).

Overall we concluded that **Score 4** and **5** respond inconsistently in certain perturbational settings, while the other scores are more robust.

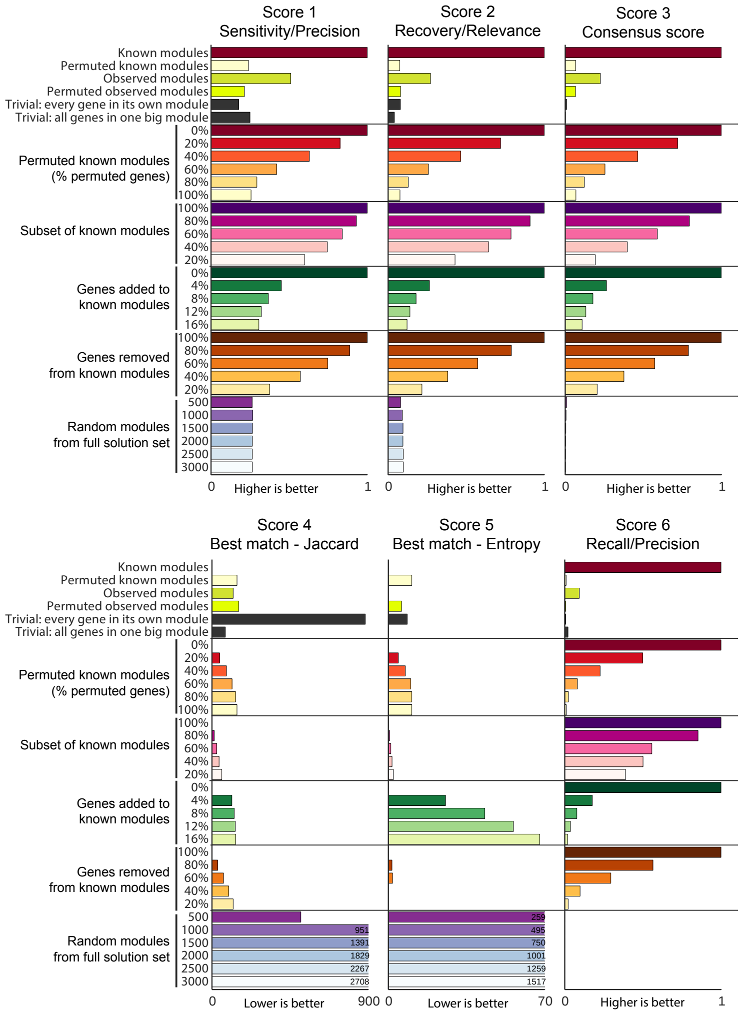

Supplementary Note 1 Figure 2: Empirical study of the robustness of several scores comparing overlapping clusters (as defined in Supplementary Note 1) in perturbational settings.
In every case, known modules (from the *E. coli* COLOMBOS dataset using the minimal co-regulation module definition) were compared to a different set of modules given in the y-axis, usually derived from the known modules but with a subset of genes permuted, a subset of modules selected or some random genes added or removed from the modules. As a reference we also give the performance of the modules detected by affinity propagation (clustering methods I) at optimal parameter settings.

Finally, we tested whether these scores can better handle both overlap and non-exhaustive cluster assignment using the test cases from **Supplementary Note 1 Figure 1**. We found that only **Score 1** still had problems regarding overlap in a subset of cases. The other scores (**Score 2**, **3** and **6**) all performed well according to our test cases. Together with the strong theoretical background of Score 6 [58] and several examples of studies where Score 2 has been successfully applied to compare biclustering methods [40, 44, 59], we chose **Score 2** and **Score 6** for the main evaluation study. The score on the other metrics, together with the three most popular classical clustering evaluation measures are given in **Supplementary Figure 8**.

	Known modules without overlap						Known modules with overlap					
	1	2	3	4	5	6	7	8	9	10	11	12
Sensitivity (Score 1a) [62]	1.00	0.50	0.75	1.00	1.00	1.00	1.00	0.83	0.75	0.58	1.00	1.00
Specificity (Score 1b) [62]	1.00	1.00	1.00	0.76	1.00	0.83	1.00	1.00	1.00	1.00	1.00	0.95
F-measure (Score 1) [62]	1.00	0.67	0.86	0.86	1.00	0.91	1.00	0.91	0.86	0.74	1.00	0.98
Recovery (Score 2a) [59]	1.00	0.50	0.75	0.76	1.00	1.00	1.00	0.83	0.62	0.58	1.00	1.00
Relevance (Score 2b) [59]	1.00	1.00	0.75	0.76	0.71	0.83	1.00	0.83	1.00	0.56	0.92	0.95
F-measure (Score 2) [59]	1.00	0.67	0.75	0.76	0.83	0.91	1.00	0.83	0.77	0.57	0.96	0.98
Consensus (Score 3) [38]	1.00	0.50	0.75	0.76	0.50	0.67	1.00	0.83	0.50	0.39	0.67	0.67
Recall (Score 6a) [64]	1.00	0.75	0.34	0.81	1.00	1.00	1.00	0.56	0.64	0.30	1.00	1.00
Precision (Score 6b) [64]	1.00	0.75	0.44	0.60	0.91	0.70	1.00	0.83	0.75	0.58	0.87	0.50
F-measure (Score 6) [64]	1.00	0.75	0.39	0.69	0.95	0.82	1.00	0.67	0.69	0.40	0.93	0.67
F-measure (Score 2 and 6)	1.00	0.71	0.51	0.73	0.89	0.86	1.00	0.74	0.73	0.47	0.94	0.80

Supplementary Note 1 Table 2: Comparison of different measures for comparing two sets of modules, based on the test cases described in Supplementary Note 1 Figure 1.
Unlike the metrics in **Supplementary Note 1 Table 1**, all metrics have been developed for overlapping and non-exhaustive sets of modules. Compared with the score on test cases 1 and 7 (grey), a good score should consistently score lower on cases 2-6 and 8-12 (green).

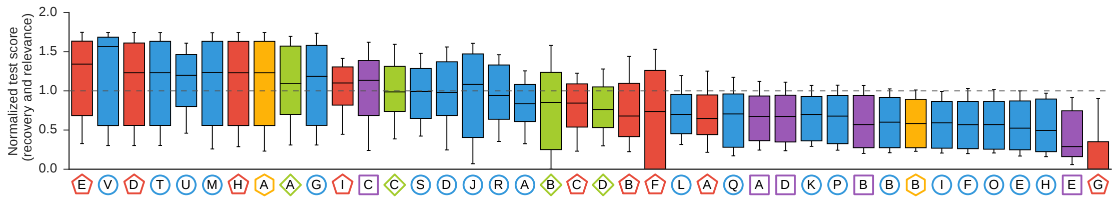

Supplementary Note 1 Figure 3: Comparing known and observed modules on human datasets. Shown are the distribution of normalized test scores when comparing known modules with observed modules on three human datasets (GTEX, TCGA and SEEK GPL) using the Recovery and Relevance scores. Known modules were extracted from the regulatory circuits networks [66] at different cutoffs using the minimal coregulation definition (as described in the **Methods**). We found that none of the module detection methods consistently outperformed permuted known modules across the different datasets and cutoffs.

While almost all module detection methods performed better than permutations of the known modules on *E. coli*, yeast and synthetic data, we found that the performance was generally very low on human datasets, rarely reaching the performance levels of permuted modules (**Supplementary Note 1 Figure 3**). We reasoned that this was mainly because of the extremely high number of false positive interactions in current large-scale human regulatory networks due to (i) promiscuous binding, (ii) context specific regulation and (iii) the difficulty of linking binding events to the activity of a promoter and (iv) the degeneracy of binding specificity.

We therefore developed a new score (aucodds) which, instead of looking at the exact overlap between known and observed modules, will use the enrichment of known targets of a particular transcription factor within the observed modules. To calculate the aucodds score given a regulatory network and observed modules, first the enrichment of target genes is calculated for every observed module and transcriptional regulator using a Fisher's exact test. Next, after correction for multiple testing, we calculate for every regulator the best odds ratio in the modules where the regulator's target genes are enriched (q-value < 0.1). Finally, for a range of odds-ratio cutoff values the percentage of regulators with an equal or larger odds-ratio are calculated and these values are combined within a final score by calculating the area under the curve formed by the log10-cutoff values and the percentage of enriched regulators. The score therefore not only looks at whether the targets of a regulator are enriched in any of the modules, but also how strongly they are enriched.

We found the aucodds score to be more stable when false-positive interactions are added to the gold standard (**Supplementary Note 1 Figure 4a**), while Scores 2 and 6 quickly converged to the levels of permuted modules. Although this score is therefore much more robust against large number of false positive regulatory interactions, it conversely also makes the score less sensitive to false positive genes in the observed modules compared with previously described measures (**Supplementary Note 1 Figure 4b**). Nonetheless, we found the aucodds score to be highly correlated with other scores for overlapping modules on the *E. coli*, yeast and synthetic datasets across parameter settings and methods (**Supplementary Note 1 Figure 4c**).

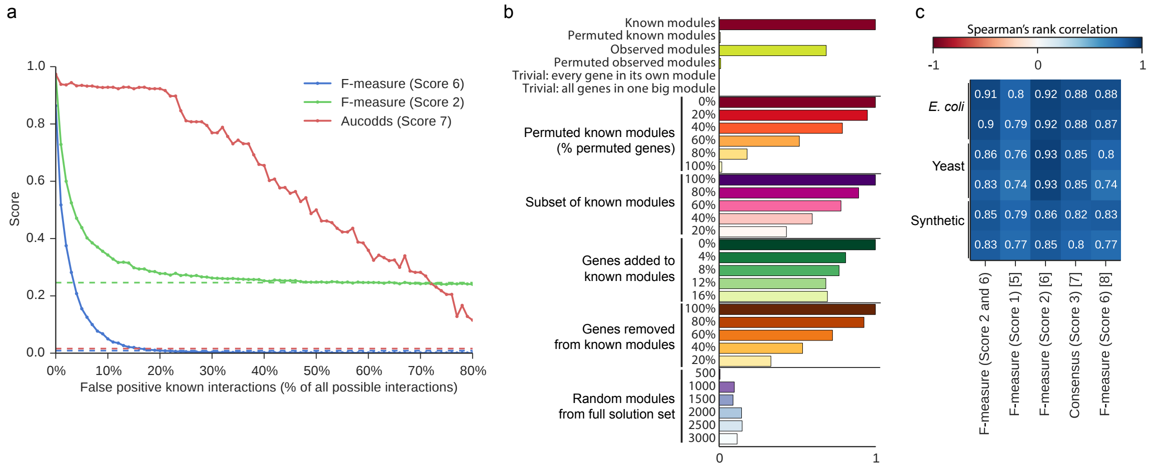

Supplementary Note 1 Figure 4: (a) The aucodds score (Score 7) decreases more slowly than other scores with increasing number of false positive regulatory interactions. **(b)** Empirical study of the robustness of aucodds score (Score 7) (as defined in **Supplementary Note 1**) in perturbational settings. See **Supplementary Note 1 Figure 2** **(c)** Spearman correlation between the aucodds score and other scores on all perturbational settings in **(b)**.

.

Supplementary Note 2: Module detection methods

Here we briefly describe every method, their implementation and the parameter settings which were varied during parameter tuning. We consider the following properties of each method:

Overlap Whether the method can assign a gene to multiple modules.

Local co-expression Whether the method can detect local co-expression in only a subset of the samples. This can either be qualitative (a sample is either part of the co-expression or it isn't) or quantitative (a sample is part of the co-expression to a certain degree).

Network inference Whether the method models the gene regulatory network.

Non-exhaustive Whether the method assigns every gene to at least one module. This usually means that the algorithm could not find sufficient evidence that some genes belonged to any module and classified their expression profiles as noise.

Deterministic Whether the output of the method is always the same between different runs (starting from a different state of the random number generator).

Co-expression Whether the genes within a module should be (locally or globally) co-expressed according to the algorithm. This is not necessarily the case with modules detected by direct network inference methods, as the expression of two target genes X and Y can be similar with a putative regulator Z even if the expression profiles X and Y are themselves independent.

Similarity measure Unless stated otherwise, the Pearson correlation coefficient was used as the default similarity measure. When an algorithm required a distance (dissimilarity) matrix, we subtracted the Pearson correlation from 2.

Standardization In gene expression datasets, absolute expression values are not always informative to assess co-expression because they can depend on things such as basal expression levels and differences in hybridization and do not necessarily implicate co-regulation. Therefore, when an algorithm intrinsically uses the Euclidean distance (such as K-means) or related geometric distance metrics, we standardized the expression matrix prior to module detection by shifting and scaling the expression profiles of every gene so that mean = 0 and standard deviation = 1. Where noted, we also standardized the expression matrix for some biclustering algorithms when it improved performance.

Parameters influencing module number Parameters directly influencing the number of modules are denoted with a †. These parameters were automatically estimated when comparing the performance of automatic module number estimation methods using cluster validity indices.

Module number estimation We distinguish three different ways the number of modules can be determined by a method: explicit methods require the number of modules to be explicitely provided by the user, implicit methods have other parameters which directly influence the number of modules while automatic methods can determine the number of modules automatically regardless of user parameters (usually based on cluster validity indices).

Clustering and biclustering subcategories We classified clustering and biclustering methods further into subcategories (see **Methods**). When methods use aspects from multiple subcategories, the category used in the main study is shown in bold.

1 Clustering

A FLAME: Fuzzy clustering by Local Approximation of MEmbership

Overlap: **yes**	Local co-expression: **no**	Network inference: **no**
Non-exhaustive: **yes**	Deterministic: **yes**	Co-expression: **yes**

Module number estimation: **implicit**

Clustering subcategories: **representative**, density, graph

The FLAME algorithm [1], based on an implementation from *code.google.com/p/flame-clustering/*. This algorithm uses similarities between the k-nearest neighbours (knn) to estimate local densities. Cluster Supporting Objects are then defined as genes with local maximum density, and are used to estimate fuzzy cluster memberships for the genes around them. We obtained crisp but potentially overlapping clusters by putting a threshold for the cluster membership ($threshold$). Expression data was standardized prior to clustering.

$knn^{\dagger}$ $\in \{1, 2, 4, 5, 7, 9, 11, 13\}$
$threshold$ $\in \{*, .01, 0.1, 0.2, 0.3, 0.5, 0.7, 0.9, 0.95, 0.99\}$
*: Every gene assigned to exactly one module with the highest fuzzy membership

B K-medoids

Overlap: **no** Local co-expression: **no** Network inference: **no**
Non-exhaustive: **no** Deterministic: **no** Co-expression: **yes**
Module number estimation: **explicit**
Clustering subcategories: **representative**

K-medoids clustering using the `pam` function in the R cluster package. This algorithm is very similar to K-means (see **F**), but uses an individual gene as the representative for a cluster instead of the mean. This makes the algorithm less sensitive to outliers and also allows an arbitrary distance matrix as input. The algorithm has one main parameter: the number of clusters (k).
$k^{\dagger}$ $\in \{25, 50, 75, ..., 275, 300\}$

C K-medoids with automatic module number estimation

Overlap: **no** Local co-expression: **no** Network inference: **no**
Non-exhaustive: **no** Deterministic: **no** Co-expression: **yes**
Module number estimation: **automatic**
Clustering subcategories: **representative**

See **B**. The k parameter was automatically estimated using cluster validity indices (cvi).
cvi $\in \{$Average silhouette width, Calinski-Harabasz index, Davis-Bouldin index, Kim-Ramakrishna$\}$

D Fuzzy c-means

Overlap: **yes** Local co-expression: **no** Network inference: **no**
Non-exhaustive: **yes** Deterministic: **no** Co-expression: **yes**
Module number estimation: **explicit**
Clustering subcategories: **representative**

The fuzzy c-means algorithm [67] (implemented in the mfuzz R package [23]) is based on k-means but it uses a membership vector for every gene which represents the degree to which a certain gene belongs to a certain cluster center. It uses a modified version of the inertia which, instead of using the distance to the nearest cluster center, will weight the Euclidean distance based on the membership vector. This weighing introduces another parameter (m). After convergence, crisp modules are obtained by placing a cutoff on the final membership vectors ($cutoff$). Expression data was standardized prior to clustering.
$k^{\dagger}$ $\in \{25, 75, 125, ..., 275, 325\}$
m $\in \{1.01, 1.02, 1.05, 1.1\}$
$cutoff$ $\in \{0.001, 0.01, 0.05, 0.1, 0.2, 0.5\}$

E Self-organizing maps

Overlap: **no** Local co-expression: **no** Network inference: **no**
Non-exhaustive: **no** Deterministic: **no** Co-expression: **yes**
Module number estimation: **explicit**
Clustering subcategories: **representative**

Self-organizing map (SOM) [68] clustering using the `som` function in the kohonen R package. Apart from grouping genes in modules, this algorithm also provides an intuitive 2D representation of all genes in the expression matrix. The algorithm embeds every gene in a two dimensional grid structure (with dimensions w by h). Every node has an associated expression profile, which is randomly initialized by sampling from the original data. Next, the algorithm iterates several times ($rlen$) over all genes. During an iteration, a gene is matched with a node based on the minimal distance between expression profiles (Euclidean distance), and the profile of the node and nodes in the neighbourhood are slightly (determined by the learning rate α) adjusted based on the expression profile of the gene. The magnitude by which the neighborhood of a node is influenced is determined by $radius$ parameter. The $topology$ of the grid (rectangular versus hexagonal) determines the number of connections between adjacent nodes. A high number of

iterations was chosen ($rlen = 500$) to make sure the algorithm reached convergence. Note that due to this, the running times given in **Figure 2** are almost certainly an overestimation.

Expression data was standardized prior to clustering.

$w = h^\dagger \quad \in \{6, 9, 12, ..., 24, 27\}$

$radius \quad \in \{0.5, 1, 1.5, 2\}$

$topology \quad \in \{\text{rectangular}, \text{hexagonal}\}$

F K-means

Overlap: **no** | Local co-expression: **no** | Network inference: **no**
Non-exhaustive: **no** | Deterministic: **no** | Co-expression: **yes**
Module number estimation: **explicit**
Clustering subcategories: **representative**

The k-means algorithm [69] (implemented in the python scikit-learn [8, 70] package, *scikit-learn.org*) is one of the oldest and most popular clustering techniques. The goal of the algorithm is, given a number of clusters k, to find the cluster centers that minimize the inertia (the sum of Euclidean distances between every gene and the nearest center). It does this by starting from k initial centers (usually randomly chosen from the data points), and iteratively optimizing the cluster centers and a cluster assignment until convergence.

The results of k-means are known to be very dependent on the random initialization. Moreover, like most clustering algorithms, k-means is not guaranteed to reach the most optimal clustering solution. Some modern implementations will therefore use a more intelligent initialization (the implementation we used will make sure that the initial cluster centers are distant from eachother [70]), and use multiple random starts (in our case 10) to finally choose the one with minimal intertia.

We standardized the expression data prior to clustering.

$k^\dagger \quad \in \{25, 50, 75, ..., 275, 300\}$

G MCL: Markov clustering

Overlap: **no** | Local co-expression: **no** | Network inference: **no**
Non-exhaustive: **no** | Deterministic: **yes** | Co-expression: **yes**
Module number estimation: **implicit**
Clustering subcategories: **graph**

The Markov clustering algorithm [4] will simulate random walks based on Markov chains, where the probability of getting to particular end node after a random walk of n steps can be easily calculated by repeatedly multiplying the transition matrix (which is a normalized version of the matrix denoting the edge weights) which is called expansion. The clustering method is based on the idea that random walks within the graph tend to stay around the nodes with which the starting node is (indirectly) strongly connected. Because this effect tends to disappear with increasing number of steps, MCL will "inflate" the weights after every expansion step by exponentiation with an $inflation$ parameter. This step will strengthen the connection between already strongly connected nodes, while the connection between lesser connected nodes are weakened. After convergence, the cluster structure can be extracted from the probability matrix. We used the pearson correlation as the input for the weighted graph, where correlations lower than a $threshold$ were set to zero.

$inflation^\dagger \quad \in \{1.4, 2, 3, 4, 6, 8, 10, 15, 20\}$

$threshold^\dagger \quad \in \{0, 0.1, ..., 1\}$

H Spectral clustering using the Pearson correlation

Overlap: **no** | Local co-expression: **no** | Network inference: **no**
Non-exhaustive: **no** | Deterministic: **no** | Co-expression: **yes**
Module number estimation: **explicit**
Clustering subcategories: **graph**

Spectral clustering [71] (implemented in the python scikit-learn [8] package, *scikit-learn.org*) using the Pearson correlation as input adjacency matrix. Essentially, this algorithm will apply dimensionality reduction on the adjacency matrix, so that genes which are connected (but not necessarily similar) will be close in the resulting subspace. Clusters are then defined within this subspace using other clustering algorithms, in this case k-means. The latter has one main parameter: the number of clusters (k).

$k^\dagger \quad \in \{25, 50, 75, ..., 275, 300\}$

I Affinity propagation

Overlap: **no** Local co-expression: **no** Network inference: **no**
Non-exhaustive: **no** Deterministic: **yes** Co-expression: **yes**
Module number estimation: **implicit**
Clustering subcategories: **graph**, representative

Affinity propagation [6] (using an implementation from the R apcluster package). Views every gene as a node in a network, and sends messages between nodes based on their affinity at a certain step in the iterative process. Starting from a similarity matrix (in our case the Pearson correlation), two types of messages (responsibility and availability) are being passed between genes. After a number of iterations, exemplars are identified by combining responsibilities and availabilities, based on an aditional preference parameter of every gene. We used a common preference value for all genes, which we varied based on the range of similarity values over all gene pairs: Preference $= \min(\text{similarities}) + (\max(\text{similarities}) - \min(\text{similarities})) * \alpha$. We left the additional damping parameter at default (0.5) because it had no impact on algorithm performance.

$\alpha^{\dagger} \in \{-3, -2.75, -2.5, ..., 0.75, 1\}$

J Spectral clustering with k-nearest neighbor graphs

Overlap: **no** Local co-expression: **no** Network inference: **no**
Non-exhaustive: **no** Deterministic: **no** Co-expression: **yes**
Module number estimation: **explicit**
Clustering subcategories: **graph**

See **H**. We used a k-nearest neighbor graph as input affinity matrix by setting $affinity$ to $nearest\_neighbors$. The number of neighbors is influenced by the knn parameter. Expression data was standardized before clustering.

$k^{\dagger} \in \{25, 50, 75, ..., 275, 300\}$
$knn \in \{10, 20, 30, 50, 70, 100\}$

K Transitivity clustering

Overlap: **yes** Local co-expression: **no** Network inference: **no**
Non-exhaustive: **no** Deterministic: **yes** Co-expression: **yes**
Module number estimation: **implicit**
Clustering subcategories: **graph**

Transitivity clustering [16] will try to remove and add edges within the co-expression graph so that all nodes within a connected component are completely connected with each other (form a clique). The algorithm starts from a similarity matrix where edges are removed if the similarity between the two nodes is lower than a $threshold$. The algorithm uses several heuristics to find the transitivity graph which minimizes the cost of removing existing edges and adding new edges. We used the fuzzy extension of the algorithm [72] which requires an additional $cutoff$ to convert the fuzzy memberships into crisp clusters.

$threshold^{\dagger} \in \{-0.5, -0.4, ..., 0.9\}$
$cutoff \in \{*, 0.001, 0.01, 0.05, 0.1, 0.2, 0.5\}$

*: Every gene assigned to exactly one module with the highest fuzzy membership

L WGCNA: Weighted Gene Co-expression Network Analysis

Overlap: **no** Local co-expression: **no** Network inference: **no**
Non-exhaustive: **yes** Deterministic: **yes** Co-expression: **yes**
Module number estimation: **implicit**
Clustering subcategories: **hierarchical**, graph

The WGCNA algorithm (implemented in the R WGCNA package [2]) is build around classical agglomerative hierarchical clustering (see **M**), but with several improvements. First, pairwise similarity values are used to construct a weighted gene co-expression network. The weights between genes are calculated based on the Pearson correlation but are transformed based on an additional "soft-thresholding" parameter to make the co-expression network more scale-free ($power$). Based on this network, topological overlap measure (TOM) are calculated for all gene pairs. The TOM between two genes is high when they are connected to similar genes within the weighted co-expression network. The advantage of this measure is that it considers the context of a gene pair when calculating the similarity between two genes, instead of only the two expression profiles in case of the Pearson correlation. Next, the algorithm performs

an agglomerative hierarchical clustering using the TOM similarity matrix and average linkage. Instead of cutting the tree at a certain height (like classical agglomerative hierarchical clustering), the WGCNA algorithm will then use a dynamic tree cutting algorithm which will make sure the obtained clusters satisfy several criteria related to cohesion and separation with other clusters [73].

The WGCNA package offers a lot of different options and parameters. To contrast the algorithm to a more classical agglomerative hierarchical clustering analysis, we used both the topological overlap measure and the dynamic tree cutting algorithm. We found that only two parameters had the major influence on performance: $power$ (used to convert the gene expression similarities to edge weights for the weighted co-expression network prior to calculated the TOM) and the $mergeCutHeight$ (used by the dynamic tree cutting algorithm to merge related clusters and therefore implicitly estimates the number of clusters).

$$power \in \{*, 1, 2, 3, ..., 10\}$$
$$mergeCutHeight^{\dagger} \in \{0.05, 0.10, 0.15, ..., 0.5\}$$

*: The power parameter automatically chosen using the `pickSoftThreshold` function

M Agglomerative hierarchical clustering

Overlap: **no** Local co-expression: **no** Network inference: **no**
Non-exhaustive: **no** Deterministic: **yes** Co-expression: **yes**
Module number estimation: **explicit**
Clustering subcategories: **hierarchical**

Agglomerative hierarchical clustering [74] (implemented in the cluster R package) builds a binary tree of all genes by starting from the leaves (each representing one gene) and progressively grouping genes in clusters until all genes are in one cluster. In every iteration, the algorithm uses a certain $linkage$ criterion to group two nodes (which can represent one or more genes) of the tree into a new node. After the tree is build, it is cut at a certain height given a desired number of modules (k).

$$linkage \in \{\text{ward, single, complete, mcquitty, median, centroid}\}$$
$$k^{\dagger} \in \{25, 50, 75, ..., 275, 300\}$$

N Hybrid hierarchical clustering

Overlap: **no** Local co-expression: **no** Network inference: **no**
Non-exhaustive: **no** Deterministic: **yes** Co-expression: **yes**
Module number estimation: **explicit**
Clustering subcategories: **hierarchical**

Hybrid hierarchical clustering [75] (implemented in the R hybridHclust package) combines both divisive and agglomerative hierarchical clustering. It will first apply a divisive hierarchical clustering algorithm but will make sure that a certain set of "mutual" clusters are not split. It will then finish the tree by applying a divisive clustering algorithm within each mutual cluster. After the tree is build, it is cut at a certain height given a desired number of modules (k).

$$k^{\dagger} \in \{25, 50, 75, ..., 275, 300\}$$

O Divisive hierarchical clustering

Overlap: **no** Local co-expression: **no** Network inference: **no**
Non-exhaustive: **no** Deterministic: **yes** Co-expression: **yes**
Module number estimation: **explicit**
Clustering subcategories: **hierarchical**

Divisive hierarchical clustering [74] (as implemented in the `diana` function in the cluster R package) starts from one large cluster containing every gene and will progressively split clusters until each cluster contains a single gene. At each iteration, it will split the cluster with the highest maximal dissimilarity between any two genes. After the tree is build, it is cut at a certain height given a desired number of modules (k).

$$k^{\dagger} \in \{25, 50, 75, ..., 275, 300\}$$

P Agglomerative hierarchical clustering with automatic module number estimation

Overlap: **no** Local co-expression: **no** Network inference: **no**
Non-exhaustive: **no** Deterministic: **yes** Co-expression: **yes**
Module number estimation: **automatic**
Clustering subcategories: **hierarchical**

See **M**. The k parameter was automatically estimated using cluster validity indices (cvi).

$cvi \quad \in$ {Average silhouette width, Calinski-Harabasz index, Davis-Bouldin index, Kim-Ramakrishna}

Q SOTA: Self-Organising Tree Algorithm

Overlap: **no** Local co-expression: **no** Network inference: **no**
Non-exhaustive: **no** Deterministic: **no** Co-expression: **yes**
Module number estimation: **explicit**
Clustering subcategories: **representative**

The Self-Organising Tree Algorithm (SOTA, implemented in the R clValid package) combines aspects from hierarchical clustering and self-organizing maps [76]. It starts from two nodes which are trained similar to self-organizing maps. Next, the most diverse node is split in two which are again trained. The splitting of nodes stops until a desired level of heterogeneity is reached within a node ($maxDiversity$), which is in the same order of magnitude as the input dissimilarities. Because the distributions of input dissimilarities depends on the dataset, we calculated the $maxDiversity$ as the α percentile of the input dissimilarities and optimized α as a parameter. Similar to regular self-organizing maps, we found that the other parameters of the algorithm had no significant effect on the results.

$\alpha^\dagger \quad \in \{0.05, 0.1, 0.2, 0.3, ..., 0.9\}$

R Density clustering

Overlap: **no** Local co-expression: **no** Network inference: **no**
Non-exhaustive: **no** Deterministic: **yes** Co-expression: **yes**
Module number estimation: **implicit**
Clustering subcategories: **density**

This recently proposed density-based clustering algorithm [25] (implemented in the R densityClust package) will calculate for every gene i two values: ρ_i represents the local density and δ_i the nearest point in a higher density region. It will then define cluster centers as genes with a high δ_i, because these genes are very dissimilar to other genes in high density regions. Finally, each gene is assigned to the cluster of its nearest neighbour of higher density. The algorithm has two parameters: ρ_c determines the minimal local density for cluster centers and δ_c the minimal distance to other nearby high density cluster centers.

${\delta_c}^\dagger \quad \in \{0.1, 0.2, 0.3, ..., 0.8\}$

${\rho_c}^\dagger \quad \in \{0.5, 1, 2, 3, ..., 15\}$

S CLICK: CLuster Identification via Connectivity Kernels

Overlap: **no** Local co-expression: **no** Network inference: **no**
Non-exhaustive: **yes** Deterministic: **yes** Co-expression: **yes**
Module number estimation: **implicit**
Clustering subcategories: **density**, graph

The CLICK algorithm [17] (implemented in the EXPANDER tool, available at *acgt.cs.tau.ac.il/expander*) will first find tight groups (kernels) of co-expressed genes. It will then add "singleton" genes to their nearest kernels. In a final post-processing step, similar kernels are merged. The tool exposes one parameter, ($homogeneity$), which adjusts the required tightness of the clusters.

$homogeneity^\dagger \quad \in \{0, 0.05, 0.1, 0.15, ..., 1\}$

T DBSCAN: Density-Based Spatial Clustering of Applications with Noise

Overlap: **no** Local co-expression: **no** Network inference: **no**
Non-exhaustive: **yes** Deterministic: **yes** Co-expression: **yes**
Module number estimation: **implicit**
Clustering subcategories: **density**, graph

This algorithm (as implemented in the fpc R package) categorizes each gene into three groups: core points have at least $MinPts$ within ϵ distance, a border point has at least one core point within ϵ distance and all other points are classified as noisy. Clusters are then defined as groups of core and border points each within ϵ distance to another gene of the cluster.

$\epsilon^{\dagger} \in \{0.05, 0.1, 0.15, ..., 0.6\}$

$MinPts^{\dagger} \in \{1, 2, 3, ..., 10\}$

U CLUES: CLUstEring based on local Shrinking

Overlap: **no** | Local co-expression: **no** | Network inference: **no**

Non-exhaustive: **no** | Deterministic: **no** | Co-expression: **yes**

Module number estimation: **automatic**

Clustering subcategories: **density**, representative

This algorithm [27] (implemented in the clues R package) first applies the Mean-shift algorithm for "shrinking" using the k-nearest neighbours for density estimation (with k as parameter). In a next "partitioning" step, clusters are defined by starting from a random starting point (gene) and iteratively moving to its closest (unvisited) point while recording the distances between every point. Cluster boundaries are then defined as big jumps in these recorded distances, based on an outlier detection procedure. The k parameter is automatically determined by iterating over several values and selecting the best one based on the average silhouette width. Datasets were standardized before applying this algorithm.

V Mean shift

Overlap: **no** | Local co-expression: **no** | Network inference: **no**

Non-exhaustive: **no** | Deterministic: **yes** | Co-expression: **yes**

Module number estimation: **implicit**

Clustering subcategories: **density**

The mean shift clustering algorithm (as implemented in the python scikit-learn [8] package, *scikit-learn.org*) will first apply kernel density estimation using, in this case, a Gaussian kernel (with a $bandwidth$ parameter). Next, points are iteratively shifted towards nearby regions of higher density until convergence. Datasets were standardized before applying this algorithm.

$bandwidth^{\dagger} \in \{*, 2.5, 5, 7.5, ..., 70\}$

*: Automatic bandwidth estimation using the scikit-learn `estimate_bandwidth` function.

2 Decomposition

Using decomposition methods for module detection consists of two steps. In the first step the expression matrix is decomposed in two or more matrices using several algorithms. One of these matrices generally contains weights for every gene and a particular component (in case of principal component analysis) or source signal (in case of independent component analysis). Another matrix contains the weights for every sample and module. In the second step, a module is extracted from every component/source signal using several postprocessing methods.

A ICA FDR (1): Independent Component Analysis followed by FDR estimation

Overlap: **yes** | Local co-expression: **yes** | Network inference: **no**

Non-exhaustive: **yes** | Deterministic: **no** | Co-expression: **yes**

Module number estimation: **explicit**

The basic goal of an independent component analysis (ICA) is to find a mixture of independent signals in data by decomposing it in two matrices: a source matrix and a mixing matrix. In the case of module detection the source matrix contains for every module the evidence that a certain gene belongs to that module. Similarly, the mixing matrix contains the individual contributions of a sample to every module.

Several algorithms have been developed for ICA mainly differing in the optimization criterion for independence and heuristics [77]. In this study we used the FastICA algorithm [78], implemented in the python scikit-learn [8] package (*www.scikit-learn.org*). This algorithm defines independence as maximizing non-Gaussianity of the individual source signals. We used the default measure of non-Gaussianity (*logcosh*). n independent source signals are found using an iterative process, whereby the weight vector of an individual source signal is randomly initialized and non-Gaussianity iteratively optimized until convergence.

Next, the source matrix with n source signals is post-processed to obtain crisp but potentially overlapping modules. Every source signal can be seen as originating from a heavy-tailed normal distribution. The goal of the post-processing step is selecting lower and higher cutoffs on the weights to identify the genes associated with these heavy-tails. We explored three post-processing techniques, all of which have been described in past studies. In the first post-processing method, the cutoffs were determined using false-discovery rate (FDR) estimation, previously described in [79]. We estimated the FDR using the fdrtool R library [36] with *cutoff.method* set to *fndr*. All genes with p-value lower than a $cutoff$ were added to a module and this cutoff was varied as a parameter denoting the compactness of the module.

$n^{\dagger} \in \{50, 100, 150, ..., 550, 600\}$
$cutoff \in \{10^{-1}, 10^{-2}, 10^{-3}, ..., 10^{-13}\}$

B ICA FDR (2): Independent Component Analysis followed by FDR estimation

Similar to **A**, but every component generates two modules depending on whether the genes have positive or negative weights.

C ICA z-score: Independent Component Analysis followed by z-scores

Overlap: **yes**	Local co-expression: **yes**	Network inference: **no**
Non-exhaustive: **yes**	Deterministic: **no**	Co-expression: **yes**
Module number estimation: **explicit**		

Similar method as **A**, but here the weights of every source signal were first standardized to z-scores. A gene was assigned to a module if its absolute z-score was higher than a *cutoff*. This post-processing procedure was previously described in [80].

$n^{\dagger} \in \{50, 100, 150, ..., 550, 600\}$
$cutoff \in \{0.5, 1, 1.5, ..., 6.5, 7\}$

D IPCA: Independent Principal Component Analysis

Overlap: **yes**	Local co-expression: **yes**	Network inference: **no**
Non-exhaustive: **yes**	Deterministic: **no**	Co-expression: **yes**
Module number estimation: **explicit**		

This method is similar to **A**, but uses the independent principal component analysis algorithm [81]. In essence, this algorithm applies the FastICA algorithm on the loading vectors of a principal component analysis. This preprocessing step should make the algorithm more robust to noise. The source signals are then again post-processed using FDR estimation.

$n^{\dagger} \in \{50, 100, 150, ..., 550, 600\}$
$cutoff \in \{10^{-1}, 10^{-2}, 10^{-3}, ..., 10^{-13}\}$

E PCA: Principal Component Analysis

Overlap: **yes**	Local co-expression: **yes**	Network inference: **no**
Non-exhaustive: **yes**	Deterministic: **yes**	Co-expression: **yes**
Module number estimation: **explicit**		

This method is similar to **C**, but uses principal component analysis (PCA) instead of FastICA, as implemented in the python scikit-learn [8] package (*scikit-learn.org*). Expression data was standardized prior to the PCA.

$n^{\dagger} \in \{25, 50, 75, ..., 275, 300\}$
$cutoff \in \{0.5, 1, 1.5, ..., 6.5, 7\}$

3 Biclustering

Biclustering methods detect biclusters, sets of genes and samples which share some local similarity in expression profile. The exact definition and optimization problem underlying this similarity is the basis of our categorization [82]. Overall, we distinguish three types of biclusters:

- Constant biclusters, in which the expression remains relatively constant
- Extreme biclusters, in which the expression of the genes is high or low in the samples of the biclusters compared to the normal expression variability of the genes.

- Biclusters with more complex co-expression patterns. These include:
  - Additive biclusters, in which the expression x_{ij} within a certain row i and column j is modeled by $x_{ij} = \mu + \alpha_i + \beta_j + \epsilon_{ij}$, where μ is the average expression in the bicluster and ϵ the error term which should be minimized.
  - Multiplicative biclusters, similar to additive biclusters but with $x_{ij} = \mu \times \alpha_i \times \beta_j + \epsilon_{ij}$.
  - Coherent evolution, in which the expression values can be ordered such that they monotonically increase along samples and/or genes, disregarding the magnitude.

  Note that constant biclusters can be seen as a special case of additive and multiplicative biclusters where respectively $\alpha_i = \beta_j = 0$ and $\alpha_i = \beta_j = 1$.

A Spectral biclustering

Overlap: **no** | Local co-expression: **yes** | Network inference: **no**

Non-exhaustive: **no** | Deterministic: **no** | Co-expression: **yes**

Module number estimation: **explicit**

Biclustering subcategories: **constant**

The goal of spectral biclustering [83] (implemented in the python scikit-learn [8] package, *www.scikit-learn.org*) is to re-order the genes and samples of the expression matrix in such a way to reveal a checkerboard structure. The expression within a "square" of the checkerboard (which can have any dimensions) is relatively constant and corresponds to a bicluster. Kluger et al. [83] show that this problem can be solved using a singular value decomposition (SVD). An SVD will decompose the expression matrix in n pairs of left and right singular vectors with accompanied eigenvalues. For a crisp partition, both the left and right eigenvectors are then clustered (n_{genes} and $n_{samples}$) using k-means. We found that the other parameters, including the normalization step prior to SVD, had minimal impact on performance. Gene expression profiles were standardized prior to biclustering.

$$n^{\dagger} \in \{10, 20, 50, 100, 200, 300, 400, 500\}$$
$$n_{genes}{}^{\dagger} \in \{10, 20, 50, 100, 200, 300, 400, 500\}$$

B ISA: Iterative Signature Algorithm

Overlap: **yes** | Local co-expression: **yes** | Network inference: **no**

Non-exhaustive: **yes** | Deterministic: **no** | Co-expression: **yes**

Module number estimation: **implicit**

Biclustering subcategories: **extreme**

The Iterative Signature Algorithm (ISA) [84–86], implemented in the isa2 R package (*www.github.com/gaborcsardi/ISA*). ISA will try to find biclusters in which the expression is extremely high or low relative to the genes and samples outside of the bicluster. ISA will first randomly generate $no.seeds$ seeds (which we fixed on 10000, 100 times more than the default). Starting from every seed, the genes and samples within the bicluster are iteratively optimized until convergence. In every iteration, the most extreme genes (samples) are selected based on a high z-score in the current samples (genes) of the bicluster. Here two parameters determine the cutoff of extreme expression: $isa.thr.col$ (samples) and $isa.thr.row$ (genes). Gene expression profiles were standardized prior to biclustering.

$$isa.thr.col^{\dagger} \in \{0.5, 1, 1.5, ..., 5\}$$
$$isa.thr.row^{\dagger} \in \{0.5, 1, 1.5, ..., 3\}$$

C QUBIC: QUalitative BIClustering algorithm

Overlap: **yes** | Local co-expression: **yes** | Network inference: **no**

Non-exhaustive: **yes** | Deterministic: **yes** | Co-expression: **yes**

Module number estimation: **implicit**

Biclustering subcategories: **extreme**, patterns

In essence, QUBIC [40] (implemented in the rqubic Bioconductor package) consists of two steps. In the first step the expression matrix is discretized to signed integers, where positive and negative integers represent respectively up- and downregulation (based on the q parameter) and 0 represents the unperturbed state. The number of discrete states depends on the $rank$ parameter, which we fixed on 1 because higher ranks always decreased performance. After discretization, QUBIC will start from two genes which are similarly up- or downregulated in a high number of conditions and expand this "seed" bicluster until the consistency level (a score of bicluster quality) falls under a user defined parameter ($tolerance$). Gene expression profiles were standardized prior to biclustering.

$q \in \{0.01, 0.06, 0.11, ..., 0.51\}$
$tolerance^{\dagger} \in \{0.3, 0.4, 0.5, ..., 1\}$

D Bi-Force

Overlap: **no**
Local co-expression: **yes**
Network inference: **no**
Non-exhaustive: **no**
Deterministic: **no**
Co-expression: **yes**
Module number estimation: **implicit**
Biclustering subcategories: **extreme**

Bi-Force is implemented in the BiCluE software (*biclue.mpi-inf.mpg.de*). The algorithm consists of four steps [87]. In the first step, the expression matrix is converted to a bipartite graph, where individual nodes represent genes or samples. A weighted edge is drawn between a gene and a sample if the gene is extremely expressed in that sample, where extreme expression depends on the clustering mode (m) parameter and a cutoff (t). In the next step, starting from an initial circular layout, nodes are re-arranged in an iterative manner so that genes and samples with a lot of (highly weighed) edges are located close to each other. The final layout is used to compute a partitioning using clustering algorithms. The biclusters are further post-processed: redundant biclsuters are merged while certain nodes are moved to an alternative bicluster if it improves the cost function of the algorithm. We standardized the expression matrix before applying this algorithm, as it greatly increased performance. Gene expression profiles were standardized prior to biclustering.
$t^{\dagger} \in \{0, 0.1, 0.2, 0.5, 0.75, 1, 1.5, 2, 5, 10\}$
$m \in \{\mathsf{o}, \mathsf{u}, \mathsf{l}, \mathsf{h}\}$

E FABIA: Factor Analysis for BIcluster Acquisition

Overlap: **yes**
Local co-expression: **yes**
Network inference: **no**
Non-exhaustive: **yes**
Deterministic: **no**
Co-expression: **yes**
Module number estimation: **explicit**
Biclustering subcategories: **patterns**

The FABIA algorithm [38] (implemented in the fabia Bioconductor package) was inspired by Plaid and is related to ICA. FABIA will model the expression matrix as the sum of n multiplicative biclusters. The algorithm ensures the sparsity of both gene and sample membership values using Laplacian priors. From a theoretical point of view, FABIA is strongly related to ICA, the main difference being that FABIA additionally requires sparseness in the sample dimension (i.e. the mixing matrix). Similarly to ICA, FABIA includes a post-processing step to detect crisp modules, which requires cutoff parameters for both the genes ($thresL$) and samples ($thresZ$). Although the algorithm has several other parameters, most of which influence the sparsity of the models, we found that tuning these had only negligible impact on performance.
$n^{\dagger} \in \{25, 50, 75, ..., 300\}$
$thresZ \in \{0.05, 0.2, 0.35, 0.5, 0.65\}$
$thresL \in \{*, 0.05, 0.2, 0.35, 0.5, 0.65\}$
*: Parameter automatically estimated by the `extractBic` function

F Plaid

Overlap: **yes**
Local co-expression: **yes**
Network inference: **no**
Non-exhaustive: **yes**
Deterministic: **no**
Co-expression: **yes**
Module number estimation: **explicit**
Biclustering subcategories: **patterns**

Plaid (implemented in the R biclust package) models the expression matrix as the sum of layers, where each layer represents an additive bicluster. The algorithm was first proposed by Lazzeroni and Owen [88] and further improved by Turner et al. [62]. The algorithm detects additive biclusters one by one. In every iteration a model is fitted by starting from a seed bicluster memberships and iteratively optimizing these and additive bicluster effects until convergence using least-squares. After fitting a layer, genes and samples are pruned based on how good the model can explain the data using two parameters($row.release$ and $col.release$). The maximal number of layers (i.e. biclusters) has to be explicitely given by the user ($max.layers$).
$col.release \in \{0, 0.2, 0.4, 0.6, 0.8, 1\}$
$row.release \in \{0, 0.2, 0.4, 0.6, 0.8, 1\}$
$max.layers^{\dagger} \in \{50, 100, 150, ..., 500\}$

G MSBE: Maximum Similarity Bicluster problem Extended

Overlap: **yes** Local co-expression: **yes** Network inference: **no**
Non-exhaustive: **yes** Deterministic: **no** Co-expression: **yes**
Module number estimation: **implicit**
Biclustering subcategories: **patterns**

The extended version of the MSBE algorithm [42] starts from a seed gene and sample and finds additive biclusters through extension and shrinking by demanding a minimal local similarity with the reference gene (γ). Two additional parameters control the tightness of the biclusters by preferring preference for small distances (β) and ignoring large distances (α). We fixed the number of random seed genes to 500 and seed samples to 20. Gene expression profiles were standardized prior to biclustering.

$\alpha \in \{0.1, 0.2, 0.3, 0.4\}$
$\beta \in \{0.2, 0.4, 0.6\}$
$\gamma \in \{0.5, 0.8, 1.1, 1.4\}$

H Cheng & Church

Overlap: **yes** Local co-expression: **yes** Network inference: **no**
Non-exhaustive: **yes** Deterministic: **no** Co-expression: **yes**
Module number estimation: **implicit**
Biclustering subcategories: **patterns**

The Cheng & Church algorithm [89] (implemented in the R biclust package) tries to find additive biclusters in the expression matrix. The algorithm will start from the full expression matrix, fit an additive model and remove those genes and samples which highly influence the residual error of the additive model. Once the error of the model reaches a threshold δ it will again add certain genes and samples with a low effect on the residual. The algorithm then finds the next bicluster by again starting from the whole expression matrix, but in which previously found biclusters are masked by random values. The algorithm has an additional α parameter which controls the number of genes and rows which can be deleted at once before recalculating the additive model. As this parameter mainly controls running time, it had only minimal effect on performance.

$\delta^{\dagger} \in \{0.0001, 0.0002, 0.0005, 0.001, ..., 0.5, 1\}$
$\alpha \in \{1.01, 1.1, 1.5\}$

I OPSM: The Order-Preserving Submatrix Problem

Overlap: **yes** Local co-expression: **yes** Network inference: **no**
Non-exhaustive: **yes** Deterministic: **yes** Co-expression: **yes**
Module number estimation: **implicit**
Biclustering subcategories: **patterns**

The OPSM algorithm [43] (implemented in the BicAT tool, *tik.ethz.ch/sop/bicat*) is the only algorithm in the evaluation study trying to find coherent evolution biclusters. Because of the combinatorial complexity of the problem of testing all possible biclusters, the algorithm will start from "partial models", in which the samples with highest and lowest expression of the bicluster are specified but not the samples inbetween. Among those partial models, it selects the best ℓ models based on the probability of find such partial models at random. Next, the algorithm expands every chosen partial model by adding one sample and again choses ℓ models among all possible expansions. Gene expression profiles were standardized prior to biclustering. While performance increased with increasing ℓ, it quickly stagnated around $\ell = 10$.

$\ell^{\dagger} \in \{1, 5, 10, 15, 20\}$

4 Direct network inference

Common to direct network inference (direct NI) methods is that they return a score for every regulator and target gene pair. To detect modules, we converted this weighed network to an unweighed one using a percentile $cutoff$ on this score and applied the three different different module definitions (minimal co-regulation, strict co-regulation and interconnected subgraphs, see **Methods** section). Finally modules were filtered on high overlap by removing the smallest of two modules if their jaccard similarity was higher than $maxoverlap$.

$cutoff^{\dagger} \in \{0.001, 0.002, 0.005, ..., 0.1, 0.2\}$
$maxoverlap \in \{0.6, 0.7, 0.8, 0.9\}$

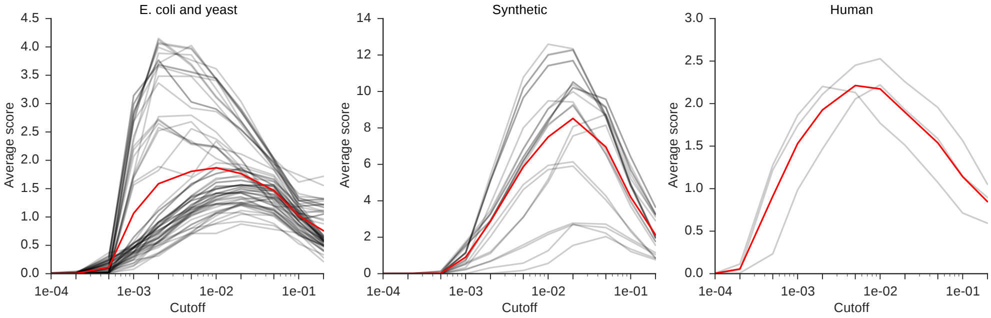

Supplementary Note 2 Figure 1: Effect of the density of the inferred regulatory network on the quality of the inferred modules. We used a percentile cutoff to convert weighted networks (as returned by direct network inference methods) to an unweighted network, which was subsequently used for graph clustering for the detection of modules. The performance of these modules generally followed a bell curve, and the most optimal density was in most cases well within the boundaries of the percentile cutoffs chosen for parameter tuning.

We assessed the performance of 3 of the top performing direct NI methods each belonging to a different category as defined in the DREAM5 evaluation study [46], together with a naive but fast network inference approach based on the Pearson correlation between regulator and target.

A GENIE3

Overlap: **yes** Local co-expression: **no** Network inference: **yes**
Non-exhaustive: **yes** Deterministic: **no** Co-expression: **no**
Module number estimation: **implicit**

The GENIE3 algorithm [47] (implemented in the genie3 R package, *montefiore.ulg.ac.be/~huynh-thu/software.html*) was the best performing algorithm in the DREAM5 network inference challenge [46]. For every target gene, GENIE3 will try to predict its expression based on regulator expression using random forests. The score for a particular regulator and target pair is then calculated using a feature importance measure.

B CLR: Context Likelihood Ratio

Overlap: **yes** Local co-expression: **no** Network inference: **yes**
Non-exhaustive: **yes** Deterministic: **yes** Co-expression: **no**
Module number estimation: **implicit**

The CLR algorithm [48] (implemented in MATLAB, *m3d.mssm.edu/network_inference.html*) first estimates mutual information similarities between all genes. It than calculates log-likelihood scores for each gene pair based on all the other mutual information scores of the two genes (i.e. its local context).

C Pearson correlation

Overlap: **yes** Local co-expression: **no** Network inference: **yes**
Non-exhaustive: **yes** Deterministic: **yes** Co-expression: **no**
Module number estimation: **implicit**

The absolute Pearson correlation coefficient between every regulator and target pair.

D TIGRESS: Trustful Inference of Gene REgulation using Stability Selection

Overlap: **yes** Local co-expression: **no** Network inference: **yes**
Non-exhaustive: **yes** Deterministic: **no** Co-expression: **no**
Module number estimation: **implicit**

TIGRESS [50] (implemented in MATLAB, *cbio.ensmp.fr/~ahaury/svn/dream5/html/index.html*) generates linear regression models to predict target gene expression based on regulator expression. It uses Least Angle Regression (LARS) to generate sparse regression coefficients. It runs LARS multiple times on slightly perturbed data to determine the regulators which are consistently selected as being predictive for target gene expression, which provides a score for every regulatory and target pair.

5 Iterative network inference

A MERLIN: Modular regulatory network learning with per gene information

Overlap: **no** | Local co-expression: **no** | Network inference: **yes**
Non-exhaustive: **yes** | Deterministic: **no** | Co-expression: **yes**
Module number estimation: **implicit**

MERLIN [53] (available at *pages.discovery.wisc.edu/~sroy/merlin*) combines strengths of both direct and module network inference algorithms [90], by making sure the genes within a module have similar regulatory programs. The algorithm starts from an initial clustering, for which we used agglomerative hierarchical clustering (with Ward's method as the linkage and number of clusters equal to $\frac{1}{10}$ the number of genes). The choice of this initial clustering algorithm and its parameters had a negligible impact on the performance. It will then predict a regulatory network between regulators and target genes using probabilistic graphical models in which p controls the number of edges in the network and r controls the influence of modules on the regulatory network. Next, the genes are clustered based on the similarity of their regulatory programs, where h determines the tightness of the resulting clusters. The two steps are repeated until convergence. Due to the computational complexity of the algorithm, especially on the yeast and human datasets, we limited the number of iterations to 10.

$h^{\dagger} \in \{0.5, 0.6, 0.7, 0.8\}$
$p \in \{-10, -8, -5, -3\}$
$r \in \{2, 4, 6, 8\}$

B Genomica

Overlap: **no** | Local co-expression: **no** | Network inference: **yes**
Non-exhaustive: **no** | Deterministic: **no** | Co-expression: **yes**
Module number estimation: **implicit**

Genomica [91] (implemented in Java, *genomica.weizmann.ac.il*) will create a so called module network, in which regulators are connected to modules instead of individual target genes. The algorithm starts from an initial clustering using a slightly adopted version of agglomerative clustering [92] (with n clusters). For every module it then predicts a regulatory program in the form of a decision tree using a probabilistic network model. Next, the algorithm determines those modules which best fit the given regulatory program. The network inference and module detection step are repeated until convergence.

$n^{\dagger} \in \{25, 50, 75, ..., 300\}$

Supplementary Note 3: Alternative similarity measures

Here we briefly describe the similarity measures evaluated in this study. Most of these are extensively described by de Siqueira Santos et al. [93], together with some practical recommendations.

Pearson correlation: The ratio between sample covariance of X and Y and the product of their standard deviations.

Distance correlation: The sample distance covariance of X and Y, normalized by the product of their distance standard deviations. The distance co-variance is calculated using the distance between individual pairs of samples, instead of a global mean in case of the regular co-variance. The distance correlation is high if X and Y to follow a similar, but potentially non-linear, curve. We did not evaluate this measure because of its excessive computation requirements (longer then 1 week on the yeast datasets).

Percentage bend correlation and **Biweight midcorrelation**: [94] Both measures use the median, rather than the mean in case of the Pearson correlation, to estimate the covariance of X and Y. This makes them more robust to outliers. Both measures are implemented in the asbio R package.

Spearman's ρ and **Kendall's** τ: These correlation measures compare the rankings, which make the measures more robust and can also detect non-linear monotonic relationships, compared to the Pearson correlation.

Mutual information: The mutual information is a symmetric measure quantifying how much the knowledge of one variable (such as the gene expression in X) tells us about another variable (gene expression in Y) and vice-versa. As such, it compares the joint probability distribution P(X,Y) with the marginal probability distributions P(X) and P(Y). We compared three different estimators for the mutual information, all implemented in the bioconductor minet package [49]: (1) the empirical estimator of Paninski [95], (2) the shrink entropy estimator Schafer and Strimmer [96] and (3) the empirical estimator corrected by Miller-Madow.

Maximal information coefficient [97]: Mutual information values are calculated by binning the gene expression values. The number of bins is selected by choosing the ones maximizing the mutual information, normalized for grid dimensions. We did not evaluate this measure because of its excessive computation requirements (longer then 1 week on the *E. coli* datasets).

Topological overlap measure [2]: The topological overlap measure quantifies whether two genes have the same gene neighbourhood in a gene co-expression network. It is therefore unique among all measures in that it not only compares the expression profiles of a gene pair, but actually takes into account the context of the co-expression among all the other genes. To create the gene co-expression network, we used the default settings in the WGCNA R package, specifically the Pearson correlation converted to a more scale-free network with $\beta = 6$.

Supplementary Note 4: Previous evaluation studies

- evaluation

  metrics:
  - Metrics should use external biological knowledge
  - Multiple evaluation metrics should be used, each assessing a different aspect of the correspondence between the gold standard and observed modules

  parameters:
  - Parameters should be optimized, moving beyond default setting while avoiding too many manual steps
  - Evaluations should control for parameter overfitting

- synthetic data

  complementary:
  - Synthetic data should be used to complement the evaluation on real data
  - Main conclusions should not be derived from synthetic data only

  realistic:
  - Synthetic data should be generated using model inspired by biology, such as gene regulatory networks
  - Synthetic data should not be biased towards certain assumptions made by a module detection method (such as a particular biclustering model)

- real data

  datasets:
  - Performance should be assessed on multiple datasets from various organisms

  gold standard:
  - Although they can certainly be informative, evaluations on real data should not uniquely depend on anecdotal evidence, but should also build upon an objective, quantitative evaluation
  - Good metrics should assess both sensitivity and precision of a method

- method coverage

  coverage:
  - Evaluations should compare state-of-the-art methods across different module detection strategies

Supplementary Note 4 Table 1: Overview of different evaluation studies of module detection methods for gene expression data and those evaluation criteria (defined above) where we think these studies do well (+ or ±) and where we think they are lacking (-).

citation		[62]	[59]	[98]	[80]	[99]	[40]	[38]	[79]	[81]	[44]	[53]	[100]	[87]
evaluation	metrics	–	±	±	–	–	±	–	–	±	±	±	+	±
	parameters	±	–	–	–	–	–	±	–	–	–	–	–	±
synthetic data	complementary	–	±	+	–	–	±	–	–	+	–	+	–	+
	realistic	–	–	–	–	–	–	–	–	–	–	±	–	–
real data	datasets	–	–	±	–	–	±	–	–	±	±	–	–	±
	gold standard	–	–	+	+	–	–	–	–	–	–	±	–	–
method coverage		–	±	–	–	–	–	±	–	–	±	–	±	±
		F	I B H M +3	M F B E +2	F C E	F H B I +2	B C M +2	E F B I A +4	L A	D E	F I B H A C E +4	A A +2	H E B I F C G +3	A F D E C H B +2

Supplementary References

[1] Limin Fu and Enzo Medico. FLAME, a novel fuzzy clustering method for the analysis of DNA microarray data. *BMC bioinformatics*, 8(1):3, 2007. ISSN 1471-2105. doi: 10.1186/1471-2105-8-3.

[2] Peter Langfelder and Steve Horvath. WGCNA: an R package for weighted correlation network analysis. *BMC bioinformatics*, 9(1):559, jan 2008. ISSN 1471-2105. doi: 10.1186/1471-2105-9-559.

[3] Peter Langfelder and Steve Horvath. Fast {R} Functions for Robust Correlations and Hierarchical Clustering. *Journal of Statistical Software*, 46(11):1–17, 2012.

[4] S M van Dongen. Graph clustering by flow simulation, feb 2001.

[5] Ulrich Bodenhofer, Andreas Kothmeier, and Sepp Hochreiter. APCluster: an R package for affinity propagation clustering. *Bioinformatics*, 27:2463–2464, 2011. doi: 10.1093/bioinformatics/btr406.

[6] Brendan J Frey and Delbert Dueck. Clustering by passing messages between data points. *Science (New York, N.Y.)*, 315(5814):972–6, feb 2007. ISSN 1095-9203. doi: 10.1126/science.1136800.

[7] Erich Schubert, Alexander Koos, Tobias Emrich, Andreas Züfle, Klaus Arthur Schmid, and Arthur Zimek. A framework for clustering uncertain data. *Proceedings of the VLDB Endowment*, 8(12):1976–1979, aug 2015. ISSN 21508097. doi: 10.14778/2824032.2824115.

[8] Fabian Pedregosa, Gaël Varoquaux, Alexandre Gramfort, Vincent Michel, Bertrand Thirion, Olivier Grisel, Mathieu Blondel, Peter Prettenhofer, Ron Weiss, Vincent Dubourg, Jake Vanderplas, Alexandre Passos, David Cournapeau, Matthieu Brucher, Matthieu Perrot, and Édouard Duchesnay. Scikit-learn: Machine Learning in Python. *Journal of Machine Learning Research*, jan 2012.

[9] Martin Maechler, Peter Rousseeuw, Anja Struyf, Mia Hubert, and Kurt Hornik. *cluster: Cluster Analysis Basics and Extensions*, 2016.

[10] Curtis Huttenhower, Mark Schroeder, Maria D Chikina, and Olga G Troyanskaya. The Sleipnir library for computational functional genomics. *Bioinformatics (Oxford, England)*, 24(13):1559–61, jul 2008. ISSN 1367-4811. doi: 10.1093/bioinformatics/btn237.

[11] Simon Barkow, Stefan Bleuler, Amela Prelic, Philip Zimmermann, and Eckart Zitzler. BicAT: a biclustering analysis toolbox. *Bioinformatics (Oxford, England)*, 22(10):1282–3, may 2006. ISSN 1367-4803. doi: 10.1093/bioinformatics/btl099.

[12] Mark Hall, Eibe Frank, Geoffrey Holmes, Bernhard Pfahringer, Peter Reutemann, and Ian H. Witten. The WEKA data mining software. *ACM SIGKDD Explorations Newsletter*, 11(1):10, nov 2009. ISSN 19310145. doi: 10.1145/1656274.1656278.

[13] Michael Reich, Ted Liefeld, Joshua Gould, Jim Lerner, Pablo Tamayo, and Jill P Mesirov. GenePattern 2.0. *Nature Genetics*, 38(5):500–501, may 2006. ISSN 1061-4036. doi: 10.1038/ng0506-500.

[14] Roberto Alonso, Francisco Salavert, Francisco Garcia-Garcia, Jose Carbonell-Caballero, Marta Bleda, Luz Garcia-Alonso, Alba Sanchis-Juan, Daniel Perez-Gil, Pablo Marin-Garcia, Ruben Sanchez, Cankut Cubuk, Marta R Hidalgo, Alicia Amadoz, Rosa D Hernansaiz-Ballesteros, Alejandro Alemán, Joaquin Tarraga, David Montaner, Ignacio Medina, and Joaquin Dopazo. Babelomics 5.0: functional interpretation for new generations of genomic data. *Nucleic acids research*, 43(W1):W117–21, jul 2015. ISSN 1362-4962. doi: 10.1093/nar/gkv384.

[15] Christian Perez-Llamas and Nuria Lopez-Bigas. Gitools: analysis and visualisation of genomic data using interactive heat-maps. *PloS one*, 6(5):e19541, jan 2011. ISSN 1932-6203. doi: 10.1371/journal.pone.0019541.

[16] Tobias Wittkop, Dorothea Emig, Sita Lange, Sven Rahmann, Mario Albrecht, John H Morris, Sebastian Böcker, Jens Stoye, and Jan Baumbach. Partitioning biological data with transitivity clustering. *Nature methods*, 7 (6):419–20, jun 2010. ISSN 1548-7105. doi: 10.1038/nmeth0610-419.

[17] R. Sharan, A. Maron-Katz, and R. Shamir. CLICK and EXPANDER: a system for clustering and visualizing gene expression data. *Bioinformatics*, 19(14):1787–1799, sep 2003. ISSN 1367-4803. doi: 10.1093/bioinformatics/btg232.

[18] Igor Ulitsky, Adi Maron-Katz, Seagull Shavit, Dorit Sagir, Chaim Linhart, Ran Elkon, Amos Tanay, Roded Sharan, Yosef Shiloh, and Ron Shamir. Expander: from expression microarrays to networks and functions. *Nature Protocols*, 5(2):303–322, feb 2010. ISSN 1754-2189. doi: 10.1038/nprot.2009.230.

[19] Ron Shamir, Adi Maron-Katz, Amos Tanay, Chaim Linhart, Israel Steinfeld, Roded Sharan, Yosef Shiloh, Ran Elkon, BM Bolstad, RA Irizarry, M Astrand, TP Speed, RA Irizarry, B Hobbs, F Collin, YD Beazer-Barclay, KJ Antonellis, U Scherf, TP Speed, MB Eisen, PT Spellman, PO Brown, D Botstein, P Tamayo, D Slonim, J Mesirov, Q Zhu, S Kitareewan, E Dmitrovsky, ES Lander, TR Golub, F Al-Shahrour, R Diaz-Uriarte, J Dopazo, S Aerts, G Thijs, B Coessens, M Staes, Y Moreau, B De Moor, R Elkon, C Linhart, R Sharan, R Shamir, Y Shiloh, R Sharan, A Maron-Katz, R Shamir, R Sharan, R Elkon, R Shamir, A Tanay, R Sharan, M Kupiec, R Shamir, J Schuchhardt, D Beule, A Malik, E Wolski, H Eickhoff, H Lehrach, H Herzel, S TP., W Huber, A von Heydebreck, H Sultmann, A Poustka, M Vingron, AE Kel, OV Kel-Margoulis, PJ Farnham, SM Bartley, E Wingender, MQ Zhang, S Tavazoie, JD Hughes, MJ Campbell, RJ Cho, GM Church, R Sharan, R Shamir, A Tanay, R Sharan, R Shamir, LF Wu, TR Hughes, AP Davierwala, MD Robinson, R Stoughton, SJ Altschuler, M Ashburner, CA Ball, JA Blake, D Botstein, H Butler, JM Cherry, AP Davis, K Dolinski, SS Dwight, JT Eppig, MA Harris, DP Hill, L Issel-Tarver, A Kasarskis, S Lewis, JC Matese, JE Richardson, M Ringwald, GM Rubin, G Sherlock, JI Murray, ML Whitfield, ND Trinklein, RM Myers, PO Brown, D Botstein, ML Whitfield, G Sherlock, AJ Saldanha, JI Murray, CA Ball, KE Alexander, JC Matese, CM Perou, MM Hurt, PO Brown, D Botstein, B Coessens, G Thijs, S Aerts, K Marchal, F De Smet, K Engelen, P Glenisson, Y Moreau, J Mathys, B De Moor, J Herrero, JM Vaquerizas, F Al-Shahrour, L Conde, A Mateos, JS Diaz-Uriarte, J Dopazo, K Hokamp, FM Roche, M Acab, ME Rousseau, B Kuo, D Goode, D Aeschliman, J Bryan, LA Babiuk, RE Hancock, FS Brinkman, KR Christie, S Weng, R Balakrishnan, MC Costanzo, K Dolinski, SS Dwight, SR Engel, B Feierbach, DG Fisk, JE Hirschman, EL Hong, L Issel-Tarver, R Nash, A Sethuraman, B Starr, CL Theesfeld, R Andrada, G Binkley, Q Dong, C Lane, M Schroeder, D Botstein, JM Cherry, N Chen, TW Harris, I Antoshechkin, C Bastiani, T Bieri, D Blasiar, K Bradnam, P Canaran, J Chan, CK Chen, WJ Chen, F Cunningham, P Davis, E Kenny, R Kishore, D Lawson, R Lee, HM Muller, C Nakamura, S Pai, P Ozersky, A Petcherski, A Rogers, A Sabo, EM Schwarz, K Van Auken, Q Wang, R Durbin, J Spieth, PW Sternberg, LD Stein, RA Drysdale, MA Crosby, W Gelbart, K Campbell, D Emmert, B Matthews, S Russo, A Schroeder, F Smutniak, P Zhang, P Zhou, M Zytkovicz, M Ashburner, A de Grey, R Foulger, G Millburn, D Sutherland, C Yamada, T Kaufman, K Matthews, A DeAngelo, RK Cook, D Gilbert, J Goodman, G Grumbling, H Sheth, V Strelets, G Rubin, M Gibson, N Harris, S Lewis, S Misra, SQ Shu, D Maglott, J Ostell, KD Pruitt, and T Tatusova. EXPANDER – an integrative program suite for microarray data analysis. *BMC Bioinformatics*, 6 (1):232, 2005. ISSN 14712105. doi: 10.1186/1471-2105-6-232.

[20] Eric Jones, Travis Oliphant, Pearu Peterson, and Others. {SciPy}: Open source scientific tools for {Python}.

[21] Daniel Müllner. fastcluster : Fast Hierarchical , Agglomerative. *Journal of Statistical Software*, 53(9):1–18, 2013. ISSN 1548-7660. doi: 10.18637/jss.v053.i09.

[22] Malika Charrad, Nadia Ghazzali, Véronique Boiteau, and Azam Niknafs. NbClust : An <i>R</i> Package for Determining the Relevant Number of Clusters in a Data Set. *Journal of Statistical Software*, 61(6):1–36, 2014. ISSN 1548-7660. doi: 10.18637/jss.v061.i06.

[23] Lokesh Kumar and Matthias E Futschik. Mfuzz: a software package for soft clustering of microarray data. *Bioinformation*, 2(1):5–7, jan 2007. ISSN 0973-2063.

[24] R Wehrens and L M C Buydens. Self- and Super-organising Maps in R: the kohonen package. *J. Stat. Softw.*, 21(5), 2007.

[25] Alex Rodriguez and Alessandro Laio. Machine learning. Clustering by fast search and find of density peaks. *Science (New York, N.Y.)*, 344(6191):1492–6, jun 2014. ISSN 1095-9203. doi: 10.1126/science.1242072.

[26] Guy Brock, Vasyl Pihur, Susmita Datta, and Somnath Datta. {clValid}: An {R} Package for Cluster Validation. *Journal of Statistical Software*, 25(4):1–22, 2008.

[27] Xiaogang Wang, Weiliang Qiu, and Ruben H. Zamar. CLUES: A non-parametric clustering method based on local shrinking. *Computational Statistics {&} Data Analysis*, 52(1):286–298, sep 2007. ISSN 01679473. doi: 10.1016/j.csda.2006.12.016.

[28] Richard S Savage, Katherine Heller, Yang Xu, Zoubin Ghahramani, William M Truman, Murray Grant, Katherine J Denby, David L Wild, M Eisen, P Spellman, P Brown, D Botstein, U Alon, N Barkai, D Notterman, K Gish, S Ybarra, D Mack, A Levine, G McLachlan, R Bean, D Peel, M Kerr, G Churchill, K Zhang, H Zhao, T Hughes, M Marton, A Jones, C Roberts, R Stoughton, C Armour, H Bennett, E Coffey, H Dai, Y He, M Kidd, A King, M Meyer, D Slade, P Lum, S Stepaniants, D Shoemaker, D Gachotte, K Chakraburtty, J Simon, M Bard, S Friend, M Levenstien, Y Yang, J Ott, J Hartigan, K Yeung, D Haynor, W Ruzzo, DJ Mackay, L Bauwens, J Rombouts, S Frühwirth-Schnatter, S Kaufmann, E Jackson, M Davy, A Doucet, W Fitzgerald, M Beaumont, B Rannala, R Neal, N Heard, C Holmes, D Stephens, D Hand, G Dimopoulos, N Heard, C Holmes, D Stephens, C Rasmussen, B de la Cruz, Z Ghahramani, DL Wild, KA Heller, Z Ghahramani, CE Rasmussen, G Brock,

V Pihur, S Datta, S Datta, W Rand, K Yeung, M Medvedovic, R Bumgarner, T Ideker, V Thorsson, J Ranish, R Christmas, J Buhler, J Eng, R Bumgarner, D Goodlett, R Aebersold, L Hood, J Yao, C Chang, M Salmi, Y Hung, A Loraine, S Roux, M de Torres-Zabala, W Truman, MH Bennett, G Lafforgue, JW Mansfield, PR Egea, L Böge, M Grant, Z Wu, R Irizarry, R Gentleman, F Martinez-Murillo, F Spencer, G Gerber, R Dowell, T Jaakkola, D Gifford, A Sidow, S Falcon, R Gentleman, S Datta, S Datta, J Jelenska, N Yao, B Vinatzer, C Wright, J Brodsky, and J Greenberg. R/BHC: fast Bayesian hierarchical clustering for microarray data. *BMC Bioinformatics*, 10(1):242, 2009. ISSN 1471-2105. doi: 10.1186/1471-2105-10-242.

[29] Eric Bonnet, Laurence Calzone, Tom Michoel, RD Hawkins, GC Hon, B Ren, LA Garraway, HR Widlund, MA Rubin, G Getz, AJ Berger, R McLendon, A Friedman, D Bigner, EG Van Meir, DJ Brat, TJ Hudson, W Anderson, A Aretz, AD Barker, C Bell, E Segal, M Shapira, A Regev, D Pe'er, D Botstein, N Friedman, S Lee, D Pe'er, A Dudley, G Church, D Koller, W Zhang, J Zhu, EE Schadt, JS Liu, SI Lee, AM Dudley, D Drubin, PA Silver, NJ Krogan, E Bonnet, M Tatari, A Joshi, T Michoel, K Marchal, E Bonnet, T Michoel, Y Van de Peer, UD Akavia, O Litvin, J Kim, F Sanchez-Garcia, D Kotliar, N Novershtern, A Regev, N Friedman, T Michoel, S Maere, E Bonnet, A Joshi, Y Saeys, A Joshi, Y Van de Peer, T Michoel, A Joshi, R De Smet, K Marchal, Y Van de Peer, T Michoel, T Michoel, R De Smet, A Joshi, Y Van de Peer, K Marchal, S Roy, I Wapinski, J Pfiffner, C French, A Socha, E Segal, CB Sirlin, C Ooi, AS Adler, J Gollub, H Zhu, H Yang, MR Owen, J Li, Z Liu, Y Pan, Q Liu, X Fu, N Novershtern, Z Itzhaki, O Manor, N Friedman, N Kaminski, I Amit, M Garber, N Chevrier, AP Leite, Y Donner, V Vermeirssen, A Joshi, T Michoel, E Bonnet, T Casneuf, N Novershtern, A Subramanian, LN Lawton, RH Mak, WN Haining, M Zhu, X Deng, T Joshi, D Xu, G Stacey, T Michoel, B Nachtergaele, S Maere, K Heymans, M Kuiper, EA Maher, FB Furnari, RM Bachoo, DH Rowitch, DN Louis, R Beroukhim, CH Mermel, D Porter, G Wei, S Raychaudhuri, TI Zack, SE Schumacher, SL Carter, AD Cherniack, G Saksena, R Beroukhim, G Getz, L Nghiemphu, J Barretina, T Hsueh, DW Parsons, S Jones, X Zhang, JCH Lin, RJ Leary, RG Verhaak, KA Hoadley, E Purdom, V Wang, Y Qi, D Hanahan, RA Weinberg, D Hanahan, RA Weinberg, I Yang, SJ Han, G Kaur, C Crane, AT Parsa, CW Brennan, RG Verhaak, A McKenna, B Campos, H Noushmehr, V Frattini, V Trifonov, JM Chan, A Castano, M Lia, SA Forbes, N Bindal, S Bamford, C Cole, CY Kok, B Vogelstein, N Papadopoulos, VE Velculescu, S Zhou, LA Diaz, MS Lawrence, P Stojanov, CH Mermel, JT Robinson, LA Garraway, A Belfiore, F Frasca, G Pandini, L Sciacca, R Vigneri, JM Cheng, JL Hiemstra, SS Schneider, A Naumova, NKV Cheung, J Wüstehube, A Bartol, SS Liebler, R Brütsch, Y Zhu, O Guzeloglu-Kayisli, UA Kayisli, NM Amankulor, JR Voorhees, O Gokce, A Toninello, P Pietrangeli, U De Marchi, M Salvi, B Mondov, E Agostinelli, G Arancia, Vedova L Dalla, F Belli, M Marra, Yj Cho, P Liang, Y Wang, D Radhakrishnan, X He, DM Peehl, and C Eng. Integrative Multi-omics Module Network Inference with Lemon-Tree. *PLOS Computational Biology*, 11(2):e1003983, feb 2015. ISSN 1553-7358. doi: 10.1371/journal.pcbi.1003983.

[30] Chris Fraley, Adrian E Raftery, Thomas Brendan Murphy, and Luca Scrucca. *mclust Version 4 for R: Normal Mixture Modeling for Model-Based Clustering, Classification, and Density Estimation*. Department of Statistics, University of Washington, 2012.

[31] Chris Fraley and Adrian E Raftery. Model-based Clustering, Discriminant Analysis and Density Estimation. *Journal of the American Statistical Association*, 97:611–631, 2002.

[32] Matthew D Wilkerson and D Neil Hayes. ConsensusClusterPlus: a class discovery tool with confidence assessments and item tracking. *Bioinformatics (Oxford, England)*, 26(12):1572–3, jun 2010. ISSN 1367-4811. doi: 10.1093/bioinformatics/btq170.

[33] Kim-Anh Lê Cao, Ignacio González, and Sébastien Déjean. integrOmics: an R package to unravel relationships between two omics datasets. *Bioinformatics (Oxford, England)*, 25(21):2855–6, nov 2009. ISSN 1367-4811. doi: 10.1093/bioinformatics/btp515.

[34] Marinka Zitnik and Blaz Zupan. NIMFA: A Python Library for Nonnegative Matrix Factorization. *Journal of Machine Learning Research*, 13:849–853, 2012.

[35] Korbinian Strimmer, T Schweder, E Spjøtvoll, Y Benjamini, Y Hochberg, J Schäfer, K Strimmer, B Efron, M Langaas, BH Lindqvist, E Ferkingstad, P Broberg, JD Storey, R Tibshirani, B Efron, C Genovese, L Wassermann, CE Bonferroni, JD Storey, JD Storey, B Efron, R Tibshirani, JD Storey, V Tusher, B Efron, J Aubert, A Bar-Hen, JJ Daudin, S Robin, B Efron, W Sun, TT Cai, K Strimmer, S Pounds, C Cheng, B Efron, R Tibshirani, GJ McLachlan, RW Bean, LBT Jones, S Robin, A Bar-Hen, JJ Daudin, L Pierre, Z Guan, B Wu, H Zhao, U Grenander, T Robertson, FT Wright, RL Dykstra, S Pounds, SW Morris, BB Turnbull, B Efron, S Scheid, R Spang, J Jin, TT Cai, C Dalmasso, P Bröet, T Moreau, JG Liao, Y Lin, ZR Selvanayagam, and WJ Shih. A unified approach to false discovery rate estimation. *BMC Bioinformatics*, 9(1):303, 2008. ISSN 1471-2105. doi: 10.1186/1471-2105-9-303.

[36] Korbinian Strimmer. fdrtool: a versatile R package for estimating local and tail area-based false discovery rates. *Bioinformatics*, 24(12):1461–2, jun 2008. ISSN 1367-4811. doi: 10.1093/bioinformatics/btn209.

[37] Sebastian Kaiser, Rodrigo Santamaria, Tatsiana Khamiakova, Martin Sill, Roberto Theron, Luis Quintales, Friedrich Leisch, and Ewoud De Troyer. *biclust: BiCluster Algorithms*, 2015.

[38] Sepp Hochreiter, Ulrich Bodenhofer, Martin Heusel, Andreas Mayr, Andreas Mitterecker, Adetayo Kasim, Tatsiana Khamiakova, Suzy Van Sanden, Dan Lin, Willem Talloen, Luc Bijnens, Hinrich W H Göhlmann, Ziv Shkedy, and Djork-Arné Clevert. FABIA: factor analysis for bicluster acquisition. *Bioinformatics*, 26(12): 1520–1527, 2010.

[39] Gábor Csárdi, Zoltán Kutalik, and Sven Bergmann. Modular analysis of gene expression data with R. *Bioinformatics (Oxford, England)*, 26(10):1376–7, may 2010. ISSN 1367-4811. doi: 10.1093/bioinformatics/btq130.

[40] Guojun Li, Qin Ma, Haibao Tang, Andrew H Paterson, and Ying Xu. {QUBIC}: a qualitative biclustering algorithm for analyses of gene expression data. *Nucleic Acids Res.*, 37(15):e101, aug 2009.

[41] Peng Sun, Jiong Guo, Jan Baumbach, DA Benson, MS Boguski, DJ Lipman, J Ostell, P Sun, J Guo, J Baumbach, T Wittkop, D Emig, S Lange, S Rahmann, M Ablrecht, JH Morris, S Böcker, J Stoye, J Baumbach, S Aluru, T Wittkop, D Emig, A Truss, M Albrecht, S Böcker, J Baumbach, A Preliç, S Bleuler, P Zimmermann, A Wille, P Buhlmann, W Gruissem, L Hennig, L Thiele, E Zitzler, N Amit, J Guo, F Protti, MD da Silva, JL Szwarcfiter, RG Downey, MR Fellows, R Niedermeier, AD Johnson, CJ O, LA Hindorff, P Sethupathy, HA Junkins, EM Ramos, JP Mehta, FS Collins, TA Manolio, AC Heath, JB Whitfield, NG Martin, ML Pergadia, AM Goate, PA Lind, BP McEvoy, AJ Schrage, JD Grant, YL Chou, R Zhu, AK Henders, SE Medland, SD Gordon, EC Nelson, A Agrawal, DR Nyholt, KK Bucholz, PA Madden, GW Montgomery, PT Ellinor, KL Lunetta, NL Glazer, A Pfeufer, A Alonso, MK Chung, MF Sinner, PI de Bakker, M Mueller, SA Lubitz, E Fox, D Darbar, NL Smith, JD Smith, RB Schnabel, EZ Soliman, KM Rice, DR Van Wagoner, BM Beckmann, C van Noord, K Wang, GB Ehret, JI Rotter, SL Hazen, G Steinbeck, AV Smith, LJ Launer, TB Harris, S Makino, M Nelis, DJ Milan, S Perz, T Esko, A Kottgen, S Moebus, C Newton-Cheh, M Li, S Mohlenkamp, TJ Wang, WH Kao, RS Vasan, MM Nothen, CA MacRae, BH Stricker, A Hofman, AG Uitterlinden, D Levy, E Boerwinkle, A Metspalu, EJ Topol, A Chakravarti, V Gudnason, BM Psaty, DM Roden, T Meitinger, HE Wichmann, JC Witteman, J Barnard, DE Arking, EJ Benjamin, SR Heckbert, S Kaab, D Pillas, CJ Hoggart, DM Evans, and PF O. BiCluE - Exact and heuristic algorithms for weighted bi-cluster editing of biomedical data. *BMC Proceedings*, 7(7):D32–D37, 2011. doi: 10.1186/1753-6561-7-S7-S9.

[42] Xiaowen Liu and Lusheng Wang. Computing the maximum similarity bi-clusters of gene expression data. *Bioinformatics (Oxford, England)*, 23(1):50–56, jan 2007. ISSN 1367-4811. doi: 10.1093/bioinformatics/btl560.

[43] Amir Ben-Dor, Benny Chor, Richard Karp, and Zohar Yakhini. Discovering local structure in gene expression data: the order-preserving submatrix problem. *Journal of computational biology : a journal of computational molecular cell biology*, 10(3-4):373–84, jan 2003. ISSN 1066-5277. doi: 10.1089/10665270360688075.

[44] Kemal Eren, Mehmet Deveci, Onur Küçüktunç, and Ümit V Çatalyürek. A comparative analysis of biclustering algorithms for gene expression data. *Brief. Bioinform.*, 14(3):279–292, 2013.

[45] Parmeet Bhatia, Serge Iovleff, and Gérard Govaert. blockcluster: An R Package for Model Based Co-Clustering, 2014.

[46] Daniel Marbach, James C Costello, Robert Küffner, Nicole M Vega, Robert J Prill, Diogo M Camacho, Kyle R Allison, DREAM5 Consortium, Manolis Kellis, James J Collins, and Gustavo Stolovitzky. Wisdom of crowds for robust gene network inference. *Nat. Methods*, 9(8):796–804, aug 2012.

[47] Vân Anh Huynh-Thu, Alexandre Irrthum, Louis Wehenkel, and Pierre Geurts. Inferring regulatory networks from expression data using tree-based methods. *PLoS One*, 5(9):10, sep 2010. ISSN 1932-6203. doi: 10.1371/journal.pone.0012776.

[48] Jeremiah J Faith, Boris Hayete, Joshua T Thaden, Ilaria Mogno, Jamey Wierzbowski, Guillaume Cottarel, Simon Kasif, James J Collins, and Timothy S Gardner. Large-scale mapping and validation of Escherichia coli transcriptional regulation from a compendium of expression profiles. *PLoS Biol.*, 5(1):e8, jan 2007. ISSN 1545-7885. doi: 10.1371/journal.pbio.0050008.

[49] Patrick E Meyer, Frédéric Lafitte, and Gianluca Bontempi. minet: A R/Bioconductor package for inferring large transcriptional networks using mutual information. *BMC bioinformatics*, 9(1):461, jan 2008. ISSN 1471-2105. doi: 10.1186/1471-2105-9-461.

[50] Anne-Claire Haury, Fantine Mordelet, Paola Vera-Licona, and Jean-Philippe Vert. TIGRESS: Trustful Inference of Gene REgulation using Stability Selection. *BMC systems biology*, 6(1):145, jan 2012. ISSN 1752-0509. doi: 10.1186/1752-0509-6-145.

[51] Adam A Margolin, Ilya Nemenman, Katia Basso, Chris Wiggins, Gustavo Stolovitzky, Riccardo Favera, Andrea Califano, MB Eisen, PT Spellman, PO Brown, D Botstein, S-K Ma, EP van Someren, LF Wessels, E Backer, MJ Reinders, N Friedman, T Ideker, V Thorsson, JA Ranish, R Christmas, J Buhler, JK Eng, R Bumgarner, DR Goodlett, R Aebersold, L Hood, AJ Butte, IS Kohane, C Wiggins, I Nemenman, H Joe, I Nemenman, J Pearl, ET Janes, J Beirlant, E Dudewicz, L Gyorfi, E van der Meulen, SP Strong, R Koberle, R de Ruyter van Steveninck, W Bialek, TM Cover, JA Thomas, CK Chow, CN Liu, P Mendes, W Sha, K Ye, K Basso, AA Margolin, G Stolovitzky, U Klein, R Dalla-Favera, A Califano, D Heckerman, AJ Hartemink, DK Gifford, TS Jaakkola, RA Young, J Yu, AV Smith, PP Wang, AJ Hartemink, ED Jarvis, GF Cooper, E Herskovits, DM Chickering, P Erdos, A Renyi, AL Barabasi, R Albert, MEJ Newman, MK Yeung, J Tegner, JJ Collins, U Klein, Y Tu, GA Stolovitzky, M Mattioli, G Cattoretti, H Husson, A Freedman, G Inghirami, L Cro, L Baldini, PC Fernandez, SR Frank, L Wang, M Schroeder, S Liu, J Greene, A Cocito, B Amati, J Tegner, MK Yeung, J Hasty, JJ Collins, TS Gardner, D di Bernardo, D Lorenz, JJ Collins, J Yedidia, M Mezard, G Parizi, H Bethe, R Kikuchi, M Opper, O Winther, JS Yedidia, WT Freeman, and Y Weiss. ARACNE: An Algorithm for the Reconstruction of Gene Regulatory Networks in a Mammalian Cellular Context. *BMC Bioinformatics*, 7(Suppl 1):S7, 2006. ISSN 14712105. doi: 10.1186/1471-2105-7-S1-S7.

[52] Gary D Bader, Christopher WV Hogue, S Fields, P Uetz, L Giot, G Cagney, TA Mansfield, RS Judson, JR Knight, T Ito, T Chiba, R Ozawa, M Yoshida, M Hattori, Y Sakaki, BL Drees, B Sundin, E Brazeau, JP Caviston, GC Chen, W Guo, M Fromont-Racine, AE Mayes, A Brunet-Simon, JC Rain, A Colley, I Dix, Y Ho, A Gruhler, A Heilbut, GD Bader, L Moore, SL Adams, AC Gavin, M Bosche, R Krause, P Grandi, M Marzioch, A Bauer, D Christendat, A Yee, A Dharamsi, Y Kluger, A Savchenko, JR Cort, SK Kim, J Lund, M Kiraly, K Duke, M Jiang, JM Stuart, AH Tong, B Drees, G Nardelli, GD Bader, B Brannetti, L Castagnoli, EA Winzeler, DD Shoemaker, A Astromoff, H Liang, K Anderson, B Andre, SA Chervitz, ET Hester, CA Ball, K Dolinski, SS Dwight, MA Harris, HW Mewes, D Frishman, C Gruber, B Geier, D Haase, A Kaps, MC Costanzo, ME Crawford, JE Hirschman, JE Kranz, P Olsen, LS Robertson, GD Bader, I Donaldson, C Wolting, BF Ouellette, T Pawson, CW Hogue, I Xenarios, L Salwinski, XJ Duan, P Higney, SM Kim, D Eisenberg, T Takai-Igarashi, Y Nadaoka, T Kaminuma, E Wingender, X Chen, R Hehl, H Karas, I Liebich, V Matys, PD Karp, M Riley, M Saier, IT Paulsen, SM Paley, A Pellegrini-Toole, R Overbeek, N Larsen, GD Pusch, M D, A Wagner, DA Fell, GW Flake, S Lawrence, CL Giles, FM Coetzee, AV Goldberg, A Ng, M Jordan, Y Weiss, H Jeong, B Tombor, R Albert, ZN Oltvai, AL Barabasi, R Albert, H Jeong, AL Barabasi, AL Barabasi, R Albert, DA Fell, A Wagner, E Hartuv, R Shamir, GD Bader, CW Hogue, P Baldi, S Brunak, Y Chauvin, CA Andersen, H Nielsen, RC Robinson, K Turbedsky, DA Kaiser, JB Marchand, HN Higgs, S Choe, AE Mayes, L Verdone, P Legrain, JD Beggs, C von Mering, R Krause, B Snel, M Cornell, SG Oliver, S Fields, H Jeong, SP Mason, AL Barabasi, ZN Oltvai, S Maslov, K Sneppen, F Gonzalez, A Delahodde, T Kodadek, SA Johnston, M Bochtler, L Ditzel, M Groll, C Hartmann, R Huber, V Batagelj, A Mrvar, T Kamada, S Kawai, T Dobzhansky, KD Pruitt, and DR Maglott. An automated method for finding molecular complexes in large protein interaction networks. *BMC Bioinformatics*, 4(1):2, 2003. ISSN 14712105. doi: 10.1186/1471-2105-4-2.

[53] Sushmita Roy, Stephen Lagree, Zhonggang Hou, James A Thomson, Ron Stewart, and Audrey P Gasch. Integrated module and gene-specific regulatory inference implicates upstream signaling networks. *PLoS computational biology*, 9(10):e1003252, jan 2013. ISSN 1553-7358. doi: 10.1371/journal.pcbi.1003252.

[54] Eran Segal, Michael Shapira, Aviv Regev, Dana Pe'er, David Botstein, Daphne Koller, and Nir Friedman. Module networks: identifying regulatory modules and their condition-specific regulators from gene expression data. *Nature genetics*, 34(2):166–76, jun 2003. ISSN 1061-4036. doi: 10.1038/ng1165.

[55] Nima Aghaeepour, Greg Finak, Holger Hoos, Tim R Mosmann, Ryan Brinkman, Raphael Gottardo, and Richard H Scheuermann. Critical assessment of automated flow cytometry data analysis techniques. *Nature methods*, 10(3):228–38, mar 2013. ISSN 1548-7105. doi: 10.1038/nmeth.2365.

[56] Christian Wiwie, Jan Baumbach, and Richard Röttger. Comparing the performance of biomedical clustering methods. *Nature Methods*, 12(11):1033–8, sep 2015. ISSN 1548-7091. doi: 10.1038/nmeth.3583.

[57] E Rendón and I Abundez. Internal versus external cluster validation indexes. *International Journal of . . .*, 2011.

[58] Enrique Amigó, Julio Gonzalo, Javier Artiles, and Felisa Verdejo. A comparison of extrinsic clustering evaluation metrics based on formal constraints. *Information Retrieval*, 12(4):461–486, jul 2008. ISSN 1386-4564. doi: 10.1007/s10791-008-9066-8.

[59] Amela Prelić, Stefan Bleuler, Philip Zimmermann, Anja Wille, Peter Bühlmann, Wilhelm Gruissem, Lars Hennig, Lothar Thiele, and Eckart Zitzler. A systematic comparison and evaluation of biclustering methods for gene expression data. *Bioinformatics*, 22(9):1122–1129, may 2006. ISSN 1367-4803. doi: 10.1093/bioinformatics/btl060.

[60] A Lancichinetti, S Fortunato, and J Kertész. Detecting the overlapping and hierarchical community structure in complex networks. *New Journal of Physics*, 2009.

[61] MK Mark K. Goldberg, Mykola Hayvanovych, and Malik Magdon-Ismail. Measuring Similarity between Sets of Overlapping Clusters. In *2010 IEEE Second International Conference on Social Computing*, pages 303–308. IEEE, aug 2010. ISBN 978-1-4244-8439-3. doi: 10.1109/SocialCom.2010.50.

[62] Heather Turner, Trevor Bailey, and Wojtek Krzanowski. Improved biclustering of microarray data demonstrated through systematic performance tests. *Comput. Stat. Data Anal.*, 48(2):235–254, 2005.

[63] Breck Baldwin Amit Bagga. Entity-Based Cross-Document Coreferencing Using the Vector Space Model. In *Proceedings of the 17th international conference on Computational linguistics*, pages 79–85. Association for Computational Linguistics, 1998. doi: 10.3115/980451.980859.

[64] Henry Rosales-Méndez and Yunior Ramírez-Cruz. CICE-BCubed: A New Evaluation Measure for Overlapping Clustering Algorithms. In *Progress in Pattern Recognition, Image Analysis, Computer Vision, and Application*, volume 8258 of *Lecture Notes in Computer Science*, pages 157–164. Springer, Heidelberg, Germany, 2013. ISBN 978-3-642-41821-1. doi: 10.1007/978-3-642-41822-8_20.

[65] Peter Langfelder, Rui Luo, Michael C Oldham, and Steve Horvath. Is my network module preserved and reproducible? *PLoS computational biology*, 7(1):e1001057, jan 2011. ISSN 1553-7358. doi: 10.1371/journal.pcbi.1001057.

[66] Daniel Marbach, David Lamparter, Gerald Quon, Manolis Kellis, Zoltán Kutalik, and Sven Bergmann. Tissue-specific regulatory circuits reveal variable modular perturbations across complex diseases. *Nature Methods*, 13 (4):366–370, mar 2016. ISSN 1548-7091. doi: 10.1038/nmeth.3799.

[67] JC Bezdek, R Ehrlich, and W Full. FCM: The fuzzy c-means clustering algorithm. *Computers & Geosciences*, 10(2):191–203, 1984.

[68] T Kohonen. *Self-Organizing Maps*. Springer, New York, 1997.

[69] J A Hartigan and M A Wong. Algorithm {AS} 136: A {K-Means} Clustering Algorithm. *J. R. Stat. Soc. Ser. C Appl. Stat.*, 28(1):100–108, 1979.

[70] D Arthur and S Vassilvitskii. k-means++: The advantages of careful seeding. *Proceedings of the eighteenth annual ACM- . . .*, 2007.

[71] J Shi and J Malik. Normalized cuts and image segmentation. *Pattern Analysis and Machine Intelligence, IEEE Transactions*, 22(8):888–905, 2000.

[72] Tobias Wittkop, Sven Rahmann, Richard Röttger, Sebastian Böcker, and Jan Baumbach. Extension and Robustness of Transitivity Clustering for Protein–Protein Interaction Network Analysis. *Internet Mathematics*, 7(4):255–273, nov 2011. ISSN 1542-7951. doi: 10.1080/15427951.2011.604559.

[73] Peter Langfelder, Bin Zhang, and Steve Horvath. Defining clusters from a hierarchical cluster tree: the Dynamic Tree Cut package for R. *Bioinformatics (Oxford, England)*, 24(5):719–20, mar 2008. ISSN 1367-4811. doi: 10.1093/bioinformatics/btm563.

[74] L Kaufman and PJ Rousseeuw. *Finding groups in data: an introduction to cluster analysis*. John Wiley & Sons, Hoboken, New Jersey, USA, Hoboken, New Jersey, USA, 2009.

[75] H. Chipman. Hybrid hierarchical clustering with applications to microarray data. *Biostatistics*, 7(2):286–301, aug 2005. ISSN 1465-4644. doi: 10.1093/biostatistics/kxj007.

[76] J Herrero, A Valencia, and J Dopazo. A hierarchical unsupervised growing neural network for clustering gene expression patterns. *Bioinformatics (Oxford, England)*, 17(2):126–36, feb 2001. ISSN 1367-4803.

[77] Aapo Hyvärinen, Juha Karhunen, and Erkki Oja. *Independent Component Analysis*. John Wiley & Sons, 2004. ISBN 0471464198.

[78] A Hyvärinen. Fast and robust fixed-point algorithms for independent component analysis. *IEEE transactions on neural networks / a publication of the IEEE Neural Networks Council*, 10(3):626–34, jan 1999. ISSN 1045-9227. doi: 10.1109/72.761722.

[79] Maxime Rotival, Tanja Zeller, Philipp S Wild, Seraya Maouche, Silke Szymczak, Arne Schillert, Raphaele Castagné, Arne Deiseroth, Carole Proust, Jessy Brocheton, Tiphaine Godefroy, Claire Perret, Marine Germain, Medea Eleftheriadis, Christoph R Sinning, Renate B Schnabel, Edith Lubos, Karl J Lackner, Heidi Rossmann, Thomas Münzel, Augusto Rendon, Jeanette Erdmann, Panos Deloukas, Christian Hengstenberg, Patrick Diemert, Gilles Montalescot, Willem H Ouwehand, Nilesh J Samani, Heribert Schunkert, David-Alexandre Tregouet, Andreas Ziegler, Alison H Goodall, François Cambien, Laurence Tiret, and Stefan Blankenberg. Integrating genome-wide genetic variations and monocyte expression data reveals trans-regulated gene modules in humans. *PLoS genetics*, 7(12):e1002367, dec 2011. ISSN 1553-7404. doi: 10.1371/journal.pgen.1002367.

[80] Andrew E Teschendorff, Michel Journée, Pierre A Absil, Rodolphe Sepulchre, and Carlos Caldas. Elucidating the altered transcriptional programs in breast cancer using independent component analysis. *PLoS computational biology*, 3(8):e161, aug 2007. ISSN 1553-7358. doi: 10.1371/journal.pcbi.0030161.

[81] Fangzhou Yao, Jeff Coquery, and Kim-Anh Lê Cao. Independent Principal Component Analysis for biologically meaningful dimension reduction of large biological data sets. *BMC bioinformatics*, 13(1):24, jan 2012. ISSN 1471-2105. doi: 10.1186/1471-2105-13-24.

[82] S C Madeira and A L Oliveira. Biclustering algorithms for biological data analysis: a survey. *IEEE/ACM Trans. Comput. Biol. Bioinform.*, 1(1):24–45, 2004.

[83] Yuval Kluger, Ronen Basri, Joseph T Chang, and Mark Gerstein. Spectral biclustering of microarray data: coclustering genes and conditions. *Genome research*, 13(4):703–16, apr 2003. ISSN 1088-9051. doi: 10.1101/gr.648603.

[84] Jan Ihmels, Gilgi Friedlander, Sven Bergmann, Ofer Sarig, Yaniv Ziv, and Naama Barkai. Revealing modular organization in the yeast transcriptional network. *Nature genetics*, 31(4):370–7, aug 2002. ISSN 1061-4036. doi: 10.1038/ng941.

[85] Sven Bergmann, Jan Ihmels, and Naama Barkai. Iterative signature algorithm for the analysis of large-scale gene expression data. *Physical review. E, Statistical, nonlinear, and soft matter physics*, 67(3 Pt 1):031902, mar 2003. ISSN 1539-3755.

[86] Jan Ihmels, Sven Bergmann, and Naama Barkai. Defining transcription modules using large-scale gene expression data. *Bioinformatics (Oxford, England)*, 20(13):1993–2003, sep 2004. ISSN 1367-4803. doi: 10.1093/bioinformatics/bth166.

[87] P. Sun, N. K. Speicher, R. Rottger, J. Guo, and J. Baumbach. Bi-Force: large-scale bicluster editing and its application to gene expression data biclustering. *Nucleic Acids Research*, 42(9):e78—-e78, mar 2014. ISSN 0305-1048. doi: 10.1093/nar/gku201.

[88] L Lazzeroni and A Owen. Plaid models for gene expression data. *Statistica sinica*, 2002.

[89] Y Cheng and G M Church. Biclustering of expression data. *Proceedings / ... International Conference on Intelligent Systems for Molecular Biology ; ISMB. International Conference on Intelligent Systems for Molecular Biology*, 8:93–103, jan 2000. ISSN 1553-0833.

[90] Riet De Smet and Kathleen Marchal. Advantages and limitations of current network inference methods. *Nat. Rev. Microbiol.*, 8(10):717–729, aug 2010.

[91] Eran Segal, Michael Shapira, Aviv Regev, Dana Pe'er, David Botstein, Daphne Koller, and Nir Friedman. Module networks: identifying regulatory modules and their condition-specific regulators from gene expression data. *Nature genetics*, 34(2):166–76, jun 2003. ISSN 1061-4036. doi: 10.1038/ng1165.

[92] Eran Segal, Dana Pe'er, Aviv Regev, and Daphne Koller. Learning module networks. *Journal of Machine Learning Research*, 6(Apr):557–588, aug 2005.

[93] Suzana de Siqueira Santos, Daniel Yasumasa Takahashi, Asuka Nakata, and André Fujita. A comparative study of statistical methods used to identify dependencies between gene expression signals. *Briefings in bioinformatics*, 15(6):906–18, nov 2014. ISSN 1477-4054. doi: 10.1093/bib/bbt051.

[94] Rand R. Wilcox. The percentage bend correlation coefficient. *Psychometrika*, 59(4):601–616, dec 1994. ISSN 0033-3123. doi: 10.1007/BF02294395.

[95] Liam Paninski. Estimation of Entropy and Mutual Information. *Neural Computation*, 15(6):1191–1253, jun 2003. ISSN 0899-7667. doi: 10.1162/089976603321780272.

[96] Juliane Schafer and Korbinian Strimmer. A Shrinkage Approach to Large-Scale Covariance Matrix Estimation and Implications for Functional Genomics. *ical Applications in Genetics and Molecular Biology*, 4(1):1175 – 1189, 2005.

[97] David N Reshef, Yakir A Reshef, Hilary K Finucane, Sharon R Grossman, Gilean McVean, Peter J Turnbaugh, Eric S Lander, Michael Mitzenmacher, and Pardis C Sabeti. Detecting novel associations in large data sets. *Science (New York, N.Y.)*, 334(6062):1518–24, dec 2011. ISSN 1095-9203. doi: 10.1126/science.1205438.

[98] Anbupalam Thalamuthu, Indranil Mukhopadhyay, Xiaojing Zheng, and George C Tseng. Evaluation and comparison of gene clustering methods in microarray analysis. *Bioinformatics (Oxford, England)*, 22(19):2405–12, oct 2006. ISSN 1367-4811. doi: 10.1093/bioinformatics/btl406.

[99] Jiajun Gu and Jun S Liu. Bayesian biclustering of gene expression data. *BMC Genomics*, 9 Suppl 1:S4, jan 2008. ISSN 1471-2164. doi: 10.1186/1471-2164-9-S1-S4.

[100] Ali Oghabian, Sami Kilpinen, Sampsa Hautaniemi, and Elena Czeizler. Biclustering methods: biological relevance and application in gene expression analysis. *PloS one*, 9(3):e90801, jan 2014. ISSN 1932-6203. doi: 10.1371/journal.pone.0090801.
